# A novel whole-cell lysate kinase assay identifies substrates of the p38 MAPK in differentiating myoblasts

**DOI:** 10.1186/2044-5040-2-5

**Published:** 2012-03-06

**Authors:** James DR Knight, Ruijun Tian, Robin EC Lee, Fangjun Wang, Ariane Beauvais, Hanfa Zou, Lynn A Megeney, Anne-Claude Gingras, Tony Pawson, Daniel Figeys, Rashmi Kothary

**Affiliations:** 1 Regenerative Medicine Program, Ottawa Hospital Research Institute, 501 Smyth Road, Ottawa, ON, K1H 8L6, Canada; 2 Department of Cellular and Molecular Medicine, University of Ottawa, 451 Smyth Road, Ottawa, ON, K1H 8M5, Canada; 3 Ottawa Institute of Systems Biology, University of Ottawa, 451 Smyth Road, Ottawa, ON, K1H 8M5, Canada; 4 Department of Biochemistry, Microbiology and Immunology, University of Ottawa, 451 Smyth Road, Ottawa, ON, K1H 8M5, Canada; 5 CAS Key Lab of Separation Sciences for Analytical Chemistry, Dalian Institute of Chemical Physics, Chinese Academy of Sciences, Dalian, 116023, China; 6 Department of Medicine, University of Ottawa, 451 Smyth Road, Ottawa, ON, K1H 8M5, Canada; 7 Samuel Lunenfeld Research Institute, Mount Sinai Hospital, 600 University Avenue, Toronto, ON, M5G 1X5, Canada; 8 Department of Molecular Genetics, University of Toronto, 1 King's College Circle, Toronto, ON, M5S 1A8, Canada; 9 Current address: Samuel Lunenfeld Research Institute, Mount Sinai Hospital, 600 University Avenue, Toronto, ON, M5G 1X5, Canada; 10 Current address: Department of Cancer Biology, Dana-Farber Cancer Institute, 450 Brookline Avenue, and Department of Genetics, Harvard Medical School, 77 Avenue Louis Pasteur, Boston, MA 02115, USA

**Keywords:** differentiation, FSBA, kinase assay, mitogen-activated protein kinase, myoblast, p38, phosphorylation, quantitative MS

## Abstract

**Background:**

The p38α mitogen-activated protein kinase (MAPK) is a critical mediator of myoblast differentiation, and does so in part through the phosphorylation and regulation of several transcription factors and chromatin remodelling proteins. However, whether p38α is involved in processes other than gene regulation during myogenesis is currently unknown, and why other p38 isoforms cannot compensate for its loss is unclear.

**Methods:**

To further characterise the involvement of p38α during myoblast differentiation, we developed and applied a simple technique for identifying relevant *in vivo *kinase substrates and their phosphorylation sites. In addition to identifying substrates for one kinase, the technique can be used *in vitro *to compare multiple kinases in the same experiment, and we made use of this to study the substrate specificities of the p38α and β isoforms.

**Results:**

Applying the technique to p38α resulted in the identification of seven *in vivo *phosphorylation sites on six proteins, four of which are cytoplasmic, in lysate derived from differentiating myoblasts. An *in vitro *comparison with p38β revealed that substrate specificity does not discriminate these two isoforms, but rather that their distinguishing characteristic appears to be cellular localisation.

**Conclusion:**

Our results suggest p38α has a novel cytoplasmic role during myogenesis and that its unique cellular localisation may be why p38β and other isoforms cannot compensate for its absence. The substrate-finding approach presented here also provides a necessary tool for studying the hundreds of protein kinases that exist and for uncovering the deeper mechanisms of phosphorylation-dependent cell signalling.

## Background

Protein kinases are well-known regulators of cell signalling and cellular behaviour that execute their function through the covalent attachment of an ATP-derived phosphate to protein substrates. To understand the function of any protein kinase on a large and cell-wide scale first requires the development of a substrate screening technique that allows for the proteins phosphorylated by a kinase of interest to be comprehensively identified, ideally in a single experiment. Although substrate-finding techniques exist, they are hindered by problems that prevent them from being easily or readily employed [1-4] and are generally limited to providing *in vitro *substrate identifications that may or may not be relevant *in vivo*. *In vivo *approaches currently available, such as that employed by Holt *et al. *[5], can associate a kinase with *in vivo *phosphorylation events, but direct phosphorylation cannot be inferred without additional experimentation. A simple technique that can identify direct *in vivo *substrates is an obvious need for the field.

The mitogen-activated protein kinase p38α is involved in several cellular processes, but its critical role during differentiation, and particularly the differentiation of myoblasts, has been a major focus. At the initiation of myoblast differentiation, p38α is known to phosphorylate several transcription factors and chromatin remodelling proteins, thereby inducing the expression of a myogenic gene program [6]. Although much is known about p38α's role in this process, it is likely very partial, and whether p38α plays an important role in other processes during myoblast differentiation, such as cell fusion or sarcomere formation, is unknown. At the same time, there are questions regarding the other p38 isoforms and their role, or lack thereof, in myogenesis. p38β is also expressed in myoblasts and is activated in the same manner as p38α, but despite having a kinase domain 75% identical to that of p38α (72% sequence identity overall), p38β is unable to compensate for the loss of p38α, even when overexpressed [7-9]. The obvious and suspected explanation is that there are critical myogenic phosphorylations specific to the α isoform, but these have yet to be discovered and whether this assumption is correct is unknown.

Here we describe a simple approach for substrate finding that can be used to identify *in vitro *and *in vivo *substrates. The technique begins with treatment of cell lysate to inactivate endogenous kinases, followed by an *in vitro *assay using an exogenous kinase of interest, and concludes with quantitative mass spectrometry (MS) to identify phosphorylation sites specific to the added kinase. By using lysate derived from vehicle- or inhibitor-treated cells, this *in vitro *approach can be simultaneously coupled with biologically relevant information to identify direct substrates regulated by the kinase of interest *in vivo*. Applying this technique to p38α with lysate from differentiating myoblasts resulted in the identification of several new *in vivo *substrates that suggest novel functions for p38α during myogenesis. We did not identify a single phosphorylation specific to the p38α isoform compared with p38β, at least in terms of *in vitro *substrate specificity, but we did see a clear difference in cellular localisation during myoblast differentiation. This leads us to propose that although the kinase domains of p38α and β likely have the same capacity to phosphorylate substrates, there are major differences in actual substrate specificity in an *in vivo *context.

## Methods

### Cell culture

C2C12 cells were grown in Dulbecco's modified Eagle's medium (DMEM) supplemented with 10% (vol/vol) foetal bovine serum with 100 U/ml penicillin, 100 μg/ml streptomycin and 250 ng/ml amphotericin B. To induce differentiation, cells were grown to 85-90% confluence, and the medium was changed to DMEM with 2% horse serum supplemented with penicillin, streptomycin and amphotericin B as described above. FLAG-tagged p38α and p38β were acquired from Addgene (Cambridge, MA, USA) [10]. Constructs were transfected into C2C12 myoblasts with Lipofectamine 2000 reagent (Invitrogen, Carlsbad, CA, USA) according to the manufacturer's instructions. To inhibit p38 activity, SB 202190 (Promega, Madison, WI, USA) solubilised in dimethyl sulphoxide (DMSO) or DMSO as control was added to differentiation media at 48 hours following the induction of differentiation, and medium with inhibitor was changed daily.

### Immunofluorescence

Cells were fixed with 4% formaldehyde and stained with the following antibodies: Flag M2 (1:1,000 dilution; Sigma-Aldrich, St Louis, MO, USA), myosin heavy chain (MyHC) (1:20 dilution; Developmental Studies Hybridoma Bank, Department of Biology, University of Iowa, Iowa City, IA, USA), Alexa Fluor 488 goat anti-mouse antibody (1:1,000 dilution; Molecular Probes/Invitrogen) and Alexa Fluor 555 goat anti-mouse antibody (1:1,000 dilution; Molecular Probes/Invitrogen). The differentiation index was calculated as the number of MyHC-positive nuclei divided by the total number of nuclei. The fusion index was quantified as the number of nuclei per MyHC-positive cell. Five fields of view at ×20 magnification were counted and averaged per replicate, with a total of three replicate experiments.

### Statistical analysis

Statistical analyses were performed using StatPlus software (AnalystSoft Inc; http://www.analystsoft.com/en/products/statplus/). The data shown are means with SD, and Student's *t*-tests were performed to determine significance for the differentiation and fusion indices.

### Western blot analysis

For Western blot analysis, cells were lysed in radioimmunoprecipitation assay (RIPA) buffer (50 mM Tris∙HCl, pH 7.5, 1% Nonidet P-40, 0.1% SDS, 150 mM NaCl, 1 mM ethylenediaminetetraacetic acid (EDTA), 50 mM NaF, 200 μM Na_3_VO_4_, 1 mM phenylmethanesulphonylfluoride (PMSF), 10 μg/ml aprotinin, 10 μg/ml leupeptin and 10 μg/ml pepstatin), and 5× Laemmli buffer (300 mM Tris, pH 6.8, 0.01% bromophenol blue, 10% SDS, 50% glycerol and 5% β-mercaptoethanol) was added to 25 μg of protein per sample to a final concentration of 1× for SDS-PAGE. For fractionation, cells were lysed in hypotonic buffer (10 mM 4-(2-hydroxyethyl)-1-piperazineethanesulphonic acid (HEPES), pH 7.5, 1.5 mM MgCl_2_, 10 mM KCl, 0.5 mM dithiothreitol (DTT), 50 mM NaF, 200 μM Na_3_VO_4_, 1 mM PMSF, 10 μg/ml aprotinin, 10 μg/ml leupeptin and 10 μg/ml pepstatin) and left on ice for 10 minutes. Cells were then passed through a 25-gauge needle three times and centrifuged at 500 *g *to pellet nuclei and unlysed cells. The supernatant was collected as whole cytoplasm. For further fractionation of the cytoplasm, the supernatant was centrifuged again at 5,000 *g *to pellet mitochondria and membrane fractions. The supernatant was then collected and centrifuged at 100,000 *g *to pellet any remaining cell particles, and the resulting supernatant was collected as cytosol. RIPA buffer was added to all fractions to a final concentration of 1× for complete lysis. Recombinant phosphorylated p38α (Millipore, Billerica, MA, USA) was dephosphorylated using λ-protein phosphatase (New England Biolabs, Inc, Ipswich, MA, USA) by adding 800 U of phosphatase to 200 ng of p38α diluted in λ-phosphatase buffer, and the sample was assayed at 30°C for 1 hour. Antibodies used for blotting were as follows: α-actinin (1:125 dilution; Abcam, Cambridge, MA, USA), COX IV (1:1,000 dilution; Abcam), GRP78 (1:500 dilution; Cell Signaling Technology, Danvers, MA, USA), lamin A/C (1:500 dilution; Abcam), MyHC (1:100 dilution; Developmental Studies Hybridoma Bank), MyoD (1:1,000 dilution; Santa Cruz Biotechnology, Santa Cruz, CA, USA), myogenin (1:100 dilution; Developmental Studies Hybridoma Bank), neural cell adhesion molecule (1:200 dilution; Abcam), p38α (1:500 dilution; Cell Signaling Technology), phospho-p38 (1:500 dilution; Abcam) and β-tubulin (1:1,000 dilution; Developmental Studies Hybridoma Bank). An Alpha Innotech HD2 imaging system (R&D Systems, Minneapolis, MN, USA) was used to quantify phospho-p38 and tubulin expression.

### FSBA treatment and substrate labelling

Cells were lysed in Nonidet P-40 buffer (50 mM Tris∙HCl, pH 7.8, 150 mM NaCl, 1% (vol/vol) Nonidet P-40, 1 mM PMSF, 10 μg/ml aprotinin, 10 μg/ml leupeptin and 10 μg/ml pepstatin). Lysate was treated at a concentration of 2 mg/ml with 20 mM 5'-4-fluorosulphonylbenzoyladenosine (FSBA) solubilised in DMSO and placed at 30°C for 1 hour. The sample was then diluted down to 1:5 with Nonidet P-40 buffer minus protease inhibitors and desalted using Millipore Amicon ultrafiltration columns with a 10 kDa molecular weight cutoff. Following concentration, the sample was diluted to 4 mg/ml with Nonidet P-40 buffer and diluted 1:2 with 2× kinase assay buffer (40 mM 3-morpholinopropane-1-sulphonic acid (MOPS), pH 7.2, 50 mM β-glycerophosphate, 10 mM ethylene glycol tetraacetic acid (EGTA), 2 mM Na_3_VO_4_, 2 mM DTT, 50 mM MgCl_2_, 400 μM cold ATP and 5 μCi [γ-^32^P]ATP). Recombinant p38α or p38β (Millipore) was added to a final concentration of 0.5% (wt/wt) total protein. Control and kinase-added samples were assayed at 30°C for 1.5 hours. For one-dimensional SDS-PAGE, 5× Laemmli buffer was added following the assay to 1×, and the sample was electrophoresed. For two-dimensional electrophoresis, 17-cm ReadyStrip immobilised pH gradient (IPG) strips (Bio-Rad Laboratories, Hercules, CA, USA) were directly rehydrated with labelled lysate diluted in rehydration buffer (7 M urea, 2 M thiourea, 4% 3-[(3-cholamidopropyl)dimethylammonio]-1-propanesulphonate (CHAPS) and 1% DTT) following the manufacturer's directions. Isoelectric focusing was performed on a PROTEAN IEF Cell (Bio-Rad Laboratories) under the following conditions: 200 V for 1 hour, 500 V for 1 hour, 5,000 V ramp for 5 hours and 5,000 V for 80,000 VH. IPG strips were then equilibrated following the manufacturer's instructions and overlaid onto a 12% SDS-PAGE gel. Following electrophoresis, gels were dried and imaged. For one-dimensional electrophoresis, 100 μg of lysate was used per reaction. For two-dimensional electrophoresis, 300 μg of lysate was used.

For *in vitro *substrate identification, assays were performed as described above with the following modifications. A quantity of 1.5 mg of lysate was treated with FSBA, the sample was desalted and 2× kinase assay buffer was added (40 mM MOPS, pH 7.2, 50 mM β-glycerophosphate, 10 mM EGTA, 2 mM Na_3_VO_4_, 2 mM DTT, 50 mM MgCl_2 _and 2 mM cold ATP). The sample was then split into three 500-μg aliquots, and 5 μg of heat-inactivated p38α was added to the control, 5 μg of active p38α was added to the second aliquot and 5 μg of active p38β was added to the third aliquot. The samples were then assayed for 3 hours at 30°C.

For *in vivo *substrate identification, assays were performed as above with the following modifications. At 48 hours of differentiation, cells were treated with 5 μM of SB 202190 or an equivalent amount of DMSO as vehicle. Twenty-four hours later the cells were lysed in Nonidet P-40 buffer. Lysate (1 mg) from DMSO-treated cells or 2× 1 mg of lysate from SB 202190-treated cells was inhibited with FSBA, the samples were desalted and 2× kinase assay buffer was added to each. A quantity of 5 μg of active p38α was added to one of the lysate aliquots from SB-treated cells. The samples were then assayed for 3 hours at 30°C.

### Dimethyl labelling

After assaying the samples, they were precipitated by methanol chloroform, then redissolved in 200 μl of 8 M urea and 50 mM Tris∙HCl, pH 8.1, with sonication. The samples were then reduced with 20 mM DTT for 1 hour at 60°C and alkylated by 100 mM iodoacetamide for 30 minutes at room temperature in the dark. Subsequently, the samples were diluted to 2 M urea with 50 mM Tris∙HCl, pH 8.1, and digested with trypsin at a protein-to-trypsin ratio of 50:1 (wt/wt) for 16 hours at 37°C. Next, the digested samples were acidified to pH 2 using 10% (vol/vol) formic acid. Dimethyl labelling of the samples was performed as reported previously [11] and is described briefly as follows. The acidified peptides were loaded onto C18 solid phase extraction (SPE) columns (50 mg of packing material). After brief washing with 50 mM sodium phosphate buffer, pH 7.5, 3 ml of light, intermediate and heavy labelling reagents were loaded onto C18 SPE columns trapped with control, p38α- and p38β-labelled samples, respectively. After being washed with 0.1% (vol/vol) formic acid, the labelled samples were eluted with 80% acetonitrile (ACN) (vol/vol) and 0.1% (vol/vol) formic acid, then dried by vacuum centrifugation.

### Phosphopeptide enrichment

Phosphopeptide enrichment by TiO_2 _was carried out as reported previously [12] with modifications. The dried samples were redissolved with 65% ACN/2% trifluoroacetic acid (TFA)/saturated glutamic acid and combined. TiO_2 _beads suspended in 65% ACN/2% TFA/saturated glutamic acid were added into the above samples with a peptide to TiO_2 _bead ratio of 1:4 (wt/wt). After being nutated for 40 minutes, the TiO_2 _beads were recovered by centrifugation and washed thoroughly with 65% ACN/2% TFA. Finally, the enriched phosphopeptides were eluted with 10% (vol/vol) NH_3_·H_2_O and dried by vacuum centrifugation.

### Online liquid chromatography tandem mass spectrometry analysis

Online liquid chromatography tandem mass spectrometry (LC-MS/MS) analysis was performed as reported previously [13,14] with modifications. The dried sample was redissolved with 0.1% formic acid and loaded onto a biphasic trap column (200 μm ID × 10 cm; 5-cm reversed phase column packed with ReproSil-Pur C18 resin (5 μm at 200 Å; Dr.Maisch GmbH, Ammerbuch-Entringen, Germany) and a 5-cm monolith strong cation exchange (SCX) column). The trapped phosphopeptides were eluted from the trap column onto a C18 tip column (75 μm ID × 20 cm, 3 μm at 200 Å; Dr.Maisch GmbH) by a series of salt washes at increasing concentrations (0, 5, 10, 15, 20, 25, 30, 35, 40, 45, 50, 55, 60, 70, 80, 90, 100, 150, 200 and 1,000 mM). Each fraction was then separated by reversed phase-based gradient elution and detected using an LTQ Orbitrap XL Fourier transform mass spectrometer (Thermo Scientific, Waltham, MA, USA). The reversed phase gradient was set as follows: 0% to 5% ACN for 2 minutes, 5% to 30% ACN for 90 minutes and 30% to 80% ACN for 5 minutes. After flushing with 80% ACN for 10 minutes, the column was equilibrated with 0.1% formic acid aqueous solution for 13 minutes. The LTQ Orbitrap XL Fourier transform mass spectrometer was operated in positive ionization mode. A voltage of 1.8 kV was applied. MS and MS/MS spectra were acquired in a data-dependent mode, and one full MS scan was followed by ten MS/MS scans. The resolution was set at 60,000 at m/z 400 after accumulation to a target value of 500,000.

### Protein identification and quantification

All MS/MS spectra in one acquired raw file were converted to a single *.mgf file using DTASuperCharge version 2.0a7 (Matrix Science, Boston, MA, USA). The *.mgf file was queried against the International Protein Index mouse database version 3.52 (EMBL-EBI; http://www.ebi.ac.uk/IPI/IPIhelp.html) using Mascot version 2.1 (Matrix Science). To evaluate the false discovery rate (FDR), reversed sequences were appended to the database. Cysteine residues were searched as a static modification of +57.0215 Da; methionine residues were searched with a variable modification of +15.9949 Da; and serine, threonine and tyrosine residues were searched with a variable modification of +79.9663 Da. Light, intermediate and heavy dimethylation of peptide amino termini and lysine residues were set as variable modifications of +28.0313 Da, +32.0564 Da and +36.0757 Da, respectively. Peptides were queried using full tryptic cleavage constraints with up to two missed cleavage sites. The mass tolerances were 7 ppm for parent masses and 0.5 Da for fragment masses. Phosphopeptides with a Mascot score ≥ 30 (rank 1, *P *≤ 0.05, bold red required) were selected and quantified (FDR < 0.01). Phosphorylation site localisation and phosphopeptide quantification were performed using a dimethyl-adapted version of MSQuant version 2.0a81. For each peptide, the putative site of phosphorylation yielding the highest posttranslational modification (PTM) score was accepted (PTM score > 13 required, as described previously [15]). Peptide ratios were obtained by calculating the extracted ion chromatograms of the light, medium and heavy forms of the peptide using the monoisotopic peaks only, and protein ratios were calculated from the average of all quantified peptides. All MSQuant outputs of the same online multidimensional separation were then imported into StatQuant version 1.2.2, and the quantified phosphopeptides were sorted together and exported.

### Substrate validation

1 μg recombinant HSP27 (Enzo Life Sciences, Farmingdale, NY, USA), recombinant Rps27 (Abnova, Taipei City, Taiwan) or recombinant Smad9 (Abnova) were diluted in 30 μl of 1× kinase assay buffer (20 mM MOPS, pH 7.2, 25 mM β-glycerophosphate, 5 mM EGTA, 1 mM Na_3_VO_4_, 1 mM DTT, 25 mM MgCl_2_, 200 μM cold ATP and 2.5 μCi [γ-^32^P]ATP). A quantity of 500 ng of p38α or p38β was then added, and the samples were assayed at 30°C for 1 hour. Laemmli buffer at 5× concentration was added to 1× to terminate the assays, then the samples were electrophoresed and the gels were dried and imaged. Peptide assays were performed similarly with 5 μg of the following peptides synthesised by Biomatik (Cambridge, ON, Canada): HSP27 S180-APLPKAVTQSAEITIPVTF, HSP27 A180-APLPKAVTQAAEITIPVTF, Rps27 S27-KHKKKRLVQSPNSYFMDVK, Rps27 A27-KHKKKRLVQAPNSYFMDVK, Smad9 T136-NPYHYQRVETPVLPPVLVP and Smad9 A136-NPYHYQRVEAPVLPPVLVP. The frequency logo was generated using WebLogo [16].

## Results

### FSBA inhibits endogenous protein kinases

**Figure 1:**
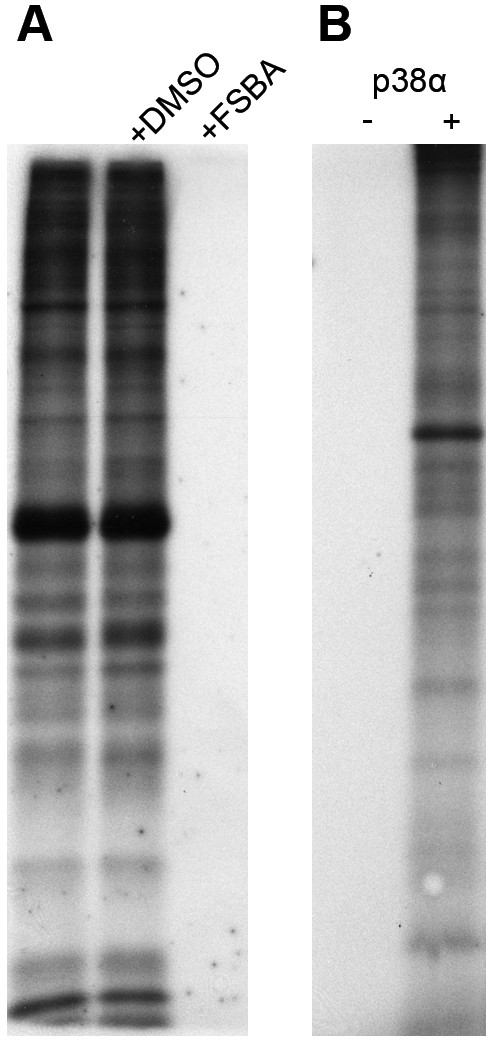
**FSBA is a pan-kinase inhibitor that allows for kinase-specific substrate labelling of cell lysate**. **(A) **C2C12 cell lysate was treated with either nothing (lane 1), dimethyl sulphoxide (DMSO) or 5'-4-fluorosulphonylbenzoyladenosine (FSBA) solubilised in DMSO. Subsequently, samples were desalted, kinase assay buffer containing ^32^P-γ-ATP was added and the samples were assayed for 1.5 hours. Treatment with FSBA abolished the labelling of endogenous protein kinase substrates. **(B) **After pretreatment of C2C12 cell lysate with FSBA, purified p38α was added with a kinase assay buffer to specifically label its substrates and visualised using one-dimensional SDS-PAGE or two-dimensional gel electrophoresis (Additional file 1 Figure S1).

A substrate-finding approach that works with cell lysate must overcome the obstacle of endogenous protein kinase activity. Lysate contains tens if not hundreds of active kinases, making it difficult to attribute individual phosphorylations that occur during a lysate-based assay to a particular kinase. 5'-4-fluorosulphonylbenzoyladenosine (FSBA) offers a simple solution. FSBA is an ATP analogue that inhibits protein kinases by occupying the ATP binding site and covalently attaching to an invariant lysine [17-20], the fully functionally conserved and so-called catalytic lysine [21]. As FSBA irreversibly occupies the ATP binding site, a bound kinase will permanently lose activity. Treatment of whole-cell C2C12 myoblast lysate with this compound can completely eliminate the endogenous kinase signal present (Figure 1A).

### Kinase-specific substrate labelling

Cell lysate treated with FSBA can be desalted to remove any unbound inhibitor and a pool of protein with no inherent kinase activity is generated. A kinase of interest can then be added with a kinase assay buffer, and any labelling that subsequently occurs is due to the added kinase as opposed to an endogenous one. To specifically label substrates of p38α, a kinase assay buffer containing [γ-^32^P]ATP was added to FSBA-treated C2C12 lysate, along with recombinant p38α, and the sample was assayed at 30°C. Substrates labelled by p38α appeared as bands following one-dimensional gel electrophoresis or as spots in two-dimensional gel electrophoresis with no contaminating signal from endogenous kinases (Figure 1B and Additional file 1 Figure S1). Although this type of approach is excellent for visualising phosphorylation, it is very difficult to identify phosphorylated proteins through spot-picking and MS. We therefore sought an alternative gel- and radioactive-free approach for identifying phosphorylated proteins.

### Quantitative MS coupled with a phosphopeptide enrichment to identify substrates

**Figure 2:**
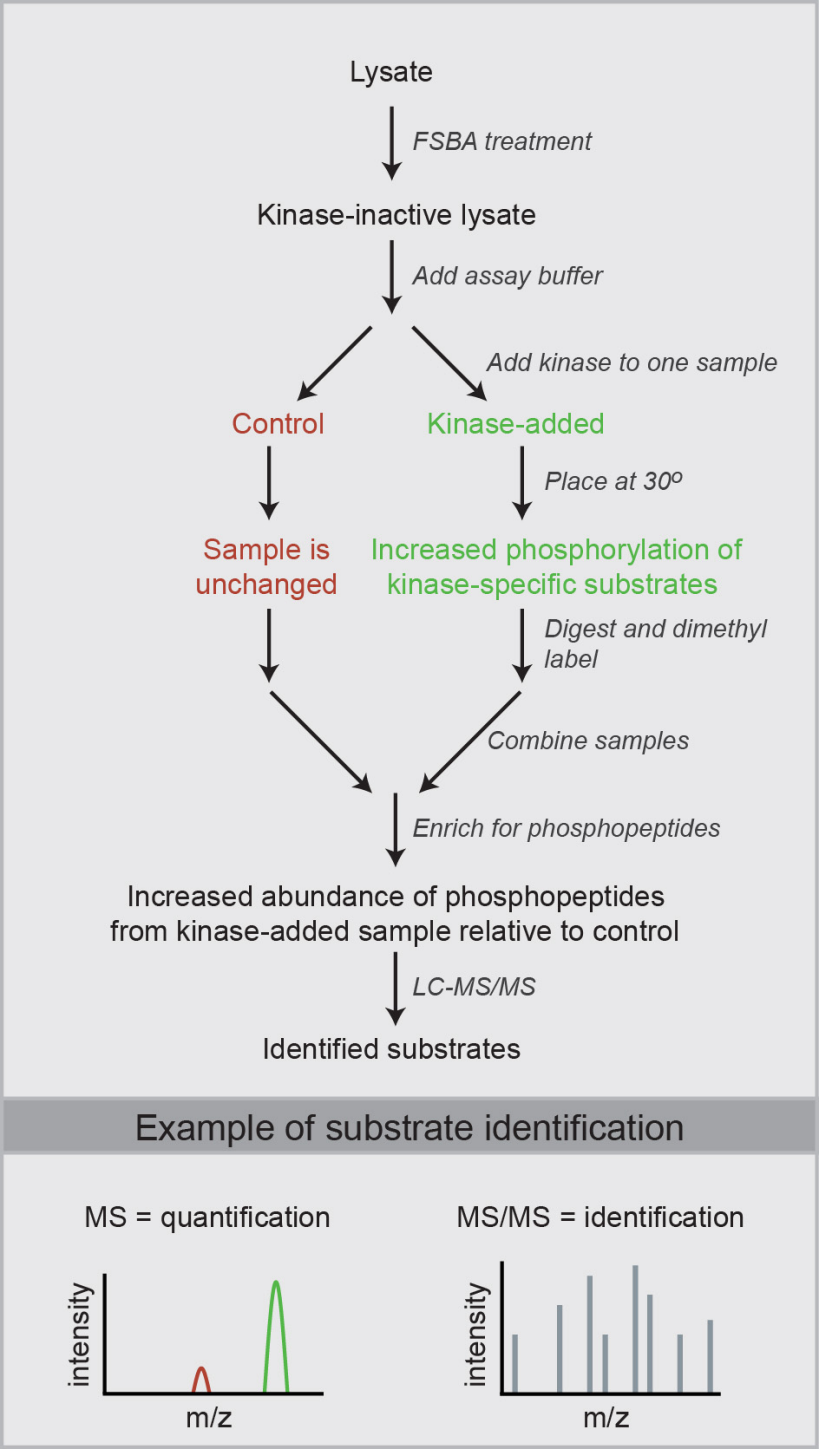
**Methodology used to identify *in vitro *kinase substrates using FSBA and quantitative mass spectrometry**. The differential tagging of peptides with dimethyl labels allows for the relative quantification and identification of phosphopeptides simultaneously in a high-mass accuracy mass spectrometer. An example/theoretical substrate identification for the added kinase (green) is shown and is based on the higher intensity of the phosphopeptide in MS.

The approach we devised to identify substrates is outlined in Figure 2. Cell lysate is treated with FSBA and desalted as described in the previous section. This is followed by the addition of a nonradioactive kinase assay buffer, and the sample is split in two, to one of which is added active kinase (kinase-added). After assaying at 30°C, the two samples are digested, and peptides from each sample are differentially tagged using isotopomeric dimethyl labels. As a final step before MS, the samples are combined and an enrichment for phosphopeptides is performed using either TiO_2 _for serine/threonine phosphorylation or a phosphotyrosine antibody for tyrosine phosphorylation. Phosphopeptides are then identified by performing LC-MS/MS, and their relative abundance between samples quantified by the differential dimethyl labelling of peptides. Phosphopeptides that are more abundant in the kinase-added sample are from proteins labelled by the added kinase during the *in vitro *assay, and by this means substrates can be identified. This approach results not only in substrate identification but also in the identification of the site of phosphorylation. Because up to three samples can be compared using dimethyl labelling, the phosphorylation profile of two kinases (plus a control) can be compared in a single experiment.

**Figure 3:**
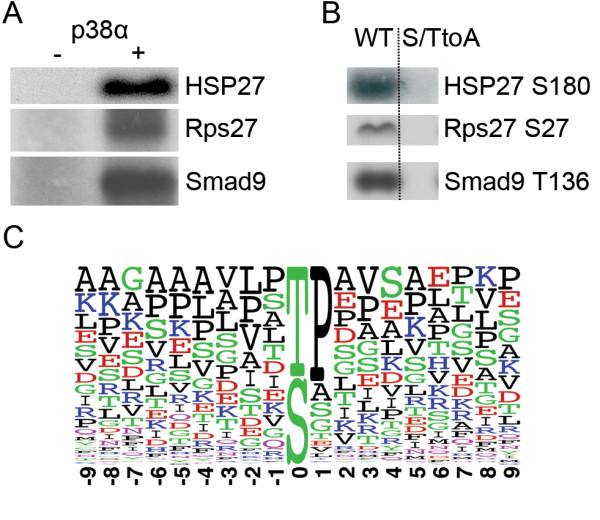
**Validation of newly discovered p38α substrates**. **(A) **Validation of three newly identified targets as *in vitro *p38α substrates was performed using full-length substrate incubated with a kinase assay buffer and p38α. **(B) **Wild-type peptides containing the phosphorylation site or peptides harbouring an S/T-to-A mutation of the phosphorylation site were incubated with p38α and a kinase assay buffer to confirm the location of the phosphorylation site. **(C) **The 158 phosphorylation sites identified were aligned, and a frequency logo was generated. p38α has a consensus phosphorylation motif of aXS/TP, where "a" is an aliphatic residue and "X" is any amino acid.

We began our initial *in vitro *substrate-finding procedure by treating 1.5 mg of C2C12 lysate with FSBA. For the assay, the sample was split into three equal parts, with active p38α and β added to the second and third samples, respectively. The rationale behind comparing p38α with p38β was to identify specific p38α phosphorylations that might explain why p38β cannot compensate for the loss of p38α in differentiating myoblasts. In total, 387 unique serine/threonine phosphopeptides were identified (Additional file 1 Table S1). A histogram of their relative abundance ratios (p38α/control) is shown in Additional file 1 Figure S2. A threefold increase in the abundance ratio was selected as a cutoff for high-confidence *in vitro *substrates as this ratio is beyond the range of inherent variability, and 158 phosphorylation sites from 94 different proteins showed at least a threefold increase in the p38α-assayed sample relative to the control. The list of p38α phosphorylation sites is presented in Additional file 1 Table S2. We identified five previously known substrates (caldesmon, histone H2B, *Psmd1*, SAKS1 and Smad3) and eighty-nine that were previously unknown. Three of these previously unknown targets were validated to determine if the technique was discovering true *in vitro *p38α substrates. Recombinant forms of these three proteins could be phosphorylated *in vitro *by p38α (Figure 3A), and a validation of peptides confirmed the site of phosphorylation (Figure 3B). After aligning substrates on their phosphorylation sites, we found that a consensus phosphorylation motif was present in many substrates, although it was not an absolute requirement (Figure 3C). The motif contains a proline immediately downstream from the target serine or threonine and an aliphatic residue two residues upstream. This motif is in agreement with that previously described for p38 [22], providing further support for our substrate-finding approach.

### Substrate specificity does not distinguish p38α and p38β

**Figure 4:**
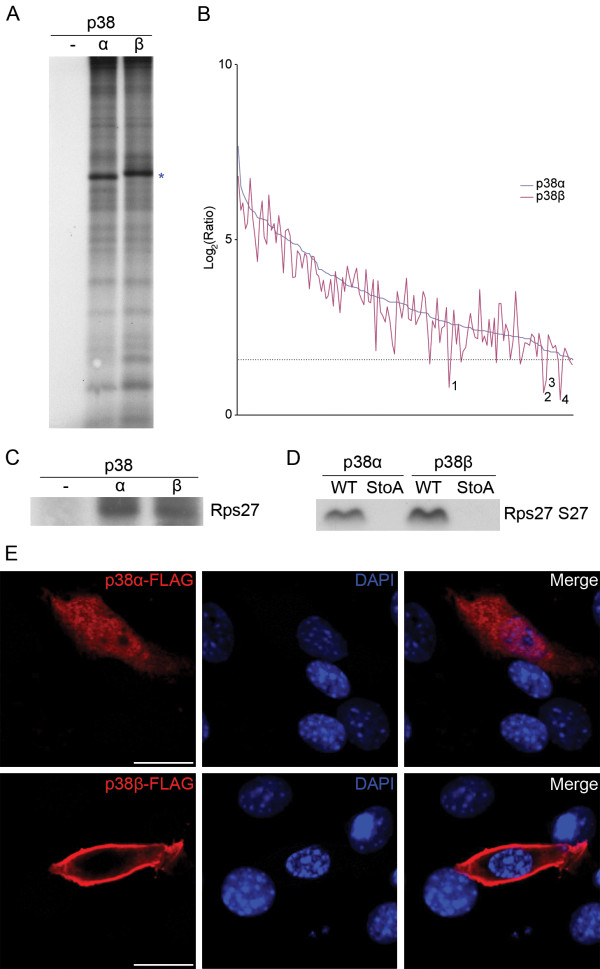
**Localisation, not substrate specificity, distinguishes p38α from p38β**. **(A) **FSBA-treated C2C12 cell lysate was incubated with a kinase assay buffer and p38α, p38β or no kinase as a control. Both isoforms produced similar banding patterns. The prominent bands marked with the asterisks in each of the p38α and p38β lanes represent autophosphorylation of the added recombinant kinase. **(B) **The quantitative values for all 158 identified p38α phosphorylations are plotted in blue on a log_2 _scale, with the corresponding values for p38β shown in red. The dashed line represents the fold cutoff for accepted substrates. Four p38β phosphorylations fell appreciably below the fold cutoff (point 2 corresponds to serine 27 of the 40S ribosomal protein S27-Rps27). **(C) **Incubation of p38α or p38β with purified Rps27 and a kinase assay buffer. **(D) **Incubation of p38α or p38β with a peptide from Rps27 containing serine 27 (or mutation of serine 27 to alanine) and a kinase assay buffer. (C) and (D) demonstrate that serine 27 of Rps27 is not a specific p38α phosphorylation site. **(E) **FLAG-tagged p38α or p38β were transfected into C2C12 cells, and at 48 hours of differentiation immunofluorescence staining for FLAG was performed. p38α has a ubiquitous distribution, whereas p38β is found solely at the cell periphery. Cells were imaged using a Zeiss LSM 510 META confocal microscope (Carl Zeiss MicroImaging GmbH, Jena, Germany). Scale bar = 20 μm.

We first assayed p38α and p38β on cell lysate using the radioactive approach and were surprised to see no obvious differences in the substrate banding patterns produced by the two isoforms (Figure 4A). Consistent with this observation, of the 158 p38α phosphorylations identified using the quantitative approach, none appeared to be specific to this isoform. The 158 quantitative values for the p38α phosphorylations are plotted from highest to lowest in Figure 4B (blue line for p38α). The red line represents the corresponding p38β values. Although p38α and p38β showed apparent differences in preference, they had very similar overall profiles. There were only four phosphorylations that fell above the threefold cutoff for p38α, whereas for p38β they were well below. However, the values were still positive for p38β, suggesting that these could be real phosphorylations but there is simply less confidence in them. To determine if these might represent specific phosphorylations, we performed *in vitro *assays with either p38α or p38β using purified substrate for one candidate. The purified substrate was the 40S ribosomal protein S27 (Rps27), and both p38 isoforms were able to phosphorylate this protein at the same site (Figure 4C and 4D). Therefore, none of the 158 phosphorylations found for p38α appear to be specific to this isoform.

If substrate specificity does not distinguish the two p38 isoforms, and yet p38β is unable to compensate for the loss of p38α, there must be an alternative characteristic that discriminates them. An obvious possibility is cellular localisation. If p38α and p38β localise differently within the cell, then p38β would simply be unable to fulfil p38α's role, even though it might have the catalytic potential to do so. To study this, we overexpressed FLAG-tagged p38α and p38β in C2C12 cells and assessed their localisation during differentiation (Figure 4E). Whereas p38α has a ubiquitous localisation pattern, p38β is found only at the periphery of the cell. p38α therefore has access to a substrate pool that p38β does not, highlighting a major reason why p38β cannot compensate for the loss of p38α.

### Identification of novel in vivo p38α substrates

Although the approach we have outlined can identify *in vitro *kinase substrates, such *in vitro *approaches will also turn up irrelevant substrates that are phosphorylated only because of the absence of the appropriate cellular context. For example, we identified just over 400 phosphorylation sites in the screen described above, 158 of which are *in vitro *p38 targets. If these numbers are representative, it suggests that p38 could be responsible for approximately 40% of all cellular phosphorylations in differentiating myoblasts, a number that seems unlikely, given that there are about 500 mouse and human kinases [23].

**Figure 5:**
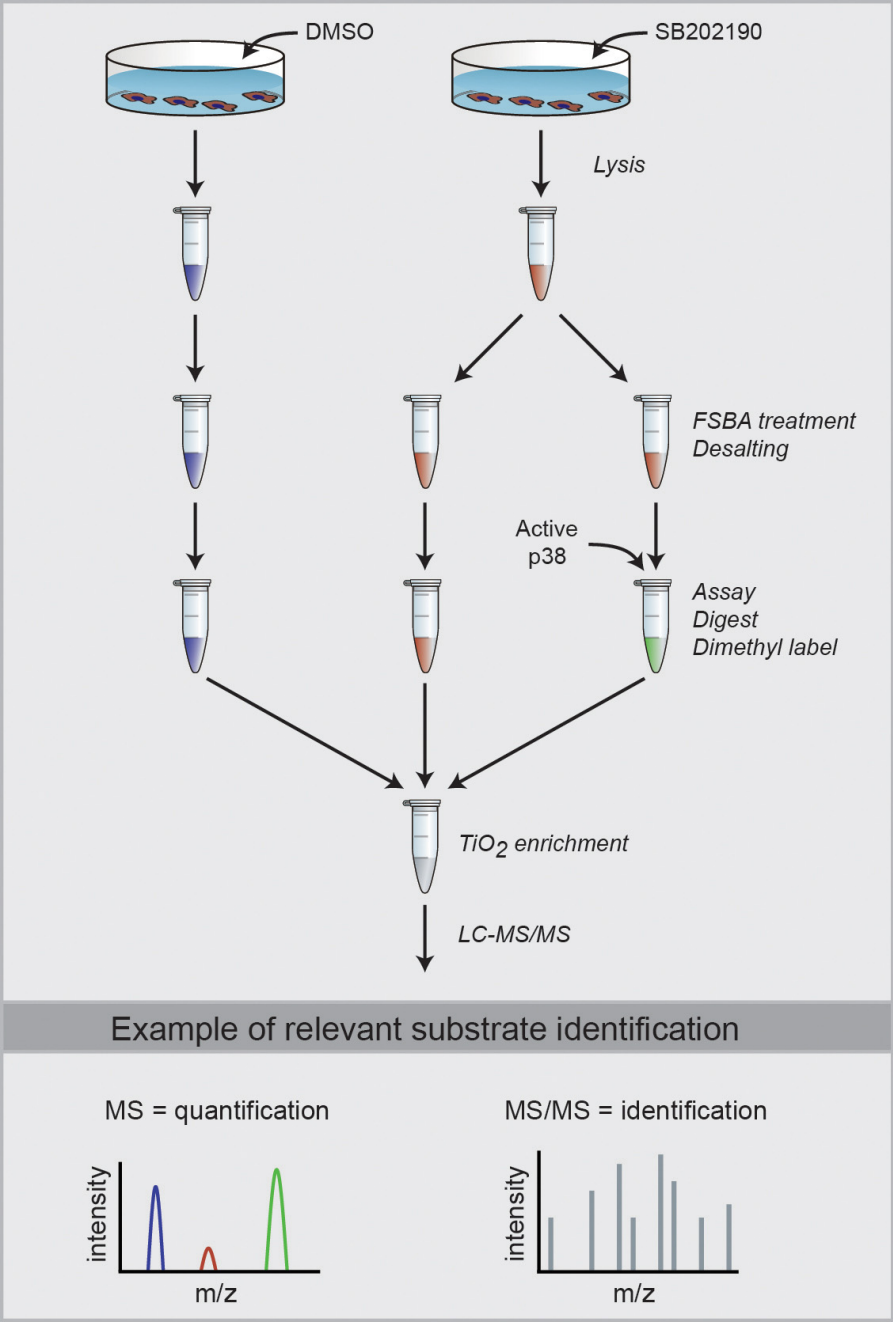
**Methodology used to identify *in vivo *kinase substrates using FSBA and quantitative MS**. *In vivo *substrates are identified based on the higher phosphopeptide intensity in MS for both the vehicle-treated (blue) and kinase-added (green) samples. A higher phosphopeptide abundance in MS for the vehicle-treated (blue) sample relative to the inhibitor-treated (red) sample indicates a phosphorylation site regulated by the kinase of interest *in vivo*. A higher phosphopeptide abundance for the kinase-added (green) sample relative to the inhibitor-treated (red) sample indicates a phosphorylation site directly targeted by the kinase *in vitro*.

To overcome the drawbacks associated with *in vitro *substrate identification, we modified the approach to allow for the identification of relevant, direct *in vivo *substrates (outlined in Figure 5). For our purposes, differentiating C2C12 cells were treated either with the p38 inhibitor SB 202190 or with DMSO as vehicle prior to lysis. SB inhibitor or vehicle treatment began at 48 hours of differentiation, and the cells were lysed 24 hours later. The 48-hour time point was chosen because of our interest in identifying novel functions for p38α during the middle stages of myoblast differentiation. Myogenic gene activation occurs within the first 48 hours of differentiation [24] and is followed by cell fusion, sarcomere formation and other processes. If differentiating C2C12 myoblasts are treated with a p38 inhibitor at 48 hours, there is a reduction in cell fusion and overall differentiation (Additional file 1 Figure S3), indicating that there is a requirement for long-term p38 activity for efficient myogenesis beyond the initial stage of myogenic gene activation, possibly both for maintaining such gene activation and for fusion-related processes. Following cell lysis, a 1-mg aliquot was taken from the lysate of vehicle-treated cells and two 1-mg aliquots were taken from the lysate of inhibitor-treated cells. All three aliquots were treated with FSBA and desalted to remove unbound FSBA and SB inhibitor, and a kinase assay buffer was added to each. Active p38α was added to one of the inhibitor-treated aliquots, and the samples were assayed at 30°C. Following the assay, the samples were digested, dimethyl-labelled and phosphopeptides enriched as described above. What we sought to identify were phosphorylation sites that decreased on inhibitor treatment and could be elevated back up by direct *in vitro *p38α labelling of SB inhibitor-treated lysate. These would be direct phosphorylation sites regulated by p38α *in vivo*.

By using this approach, we identified a total of 717 phosphorylation sites (Additional file 1 Table S3), 73 of which showed a threefold decrease following SB inhibitor treatment, and seven of these sites were direct p38α phosphorylation sites (that is, showed at least a threefold increase in the p38α-labelled sample relative to the lysate from inhibitor-treated cells). In contrast to the pure *in vitro *approach, which suggested that approximately 40% of all cellular phosphorylations might be p38 target sites, the numbers derived from the *in vivo *study suggest that the actual level is closer to about 1%. The seven *in vivo *sites (on six substrates) that we identified are listed in Table 1.

**Table 1:** Newly discovered *in vivo *p38α substrates

Genes	Protein names	Sites
*Ahnak*	Neuroblast differentiation-associated protein *AHNAK*	S4906
*Iws1*	Protein IWS1 homolog	S183, S185
*Grp78*	78 kDa glucose-regulated protein	T649
*Pgrmc*	Membrane-associated progesterone receptor component 1	S181
*Prdx6*	Peroxiredoxin 6	T93
*Ranbp2*	E3 SUMO^a ^protein ligase RanBP2	S2505

^a^SUMO, small ubiquitin-like modifier.

**Figure 6:**
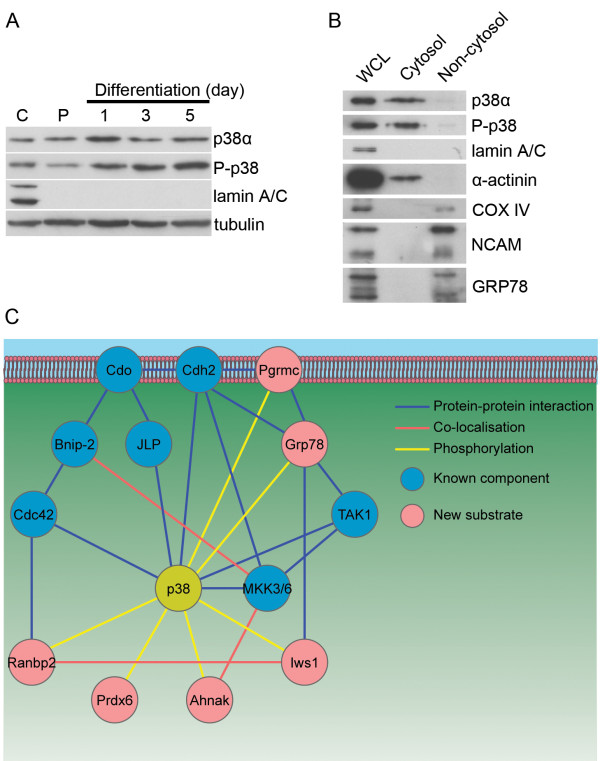
**Cytoplasmic characterization of p38α during C2C12 cell differentiation**. **(A) **C2C12 cells were fractionated over a differentiation time course. C, control whole-cell lysate; P, proliferating lysate. Western blotting for phospho-p38 shows a clear increase in the cytoplasm as differentiation proceeds. Nuclei were removed in the initial step of fractionation, and lamin A/C was used to demonstrate that there were no contaminating nuclei in cytoplasmic extracts. Quantification of phospho-p38 expression is shown in Additional file 1 Figure S4. **(B) **After 48 hours of differentiation, C2C12 cells were lysed and the cytoplasmic fraction was collected and further fractionated into cytosolic (including the cytoskeleton) and noncytosolic fractions. Nuclear marker: lamin A/C; cytoskeletal marker: α-actinin; mitochondrial marker: COX IV; membrane marker: neural cell adhesion molecule (NCAM); endoplasmic reticulum marker: GRP78. **(C) **Interconnections between the p38α activation pathway and newly discovered *in vivo *substrates. Proteins previously associated with p38α in its activation pathway are indicated as blue nodes. New substrates are indicated as red nodes. Edge colouring indicates the type of association between nodes.

The substrate Iws1 is a largely nuclear protein involved in several processes, such as chromatin remodelling and mRNA export [25,26], functions with which p38 has previously been associated. Ranbp2 localises predominantly to the nuclear pore complex and is critical for myotube formation [27]. The other four substrates are cytoplasmic proteins without known or suggested nuclear functions (Ahnak, Grp78, Pgrmc and Prdx6). On the basis of fractionations and Western blot analysis, we found that p38α was indeed present in the cytoplasm during differentiation (Figure 6A), which is in agreement with the immunofluorescence staining of FLAG-tagged p38α. The levels of phosphorylated p38, the active form, increased in the cytoplasm with differentiation (Figure 6A and Additional file 1 Figure S4), suggesting that p38α might play an important role there. Validation of the phospho-p38 antibody is shown in Additional file 1 Figure S5. Further fractionation of the cytoplasm revealed that p38α and phospho-p38 were present only in the cytosolic fraction (Figure 6B), meaning that all four of the *in vivo *cytoplasmic phosphorylation sites we identified would be accessible to p38α. Using Scansite [28], we found that five of the six substrates contained one or more predicted D domains, MAPK docking domains found on many *in vivo *substrates (D domain sites and scores are listed in Additional file 1 Table S4). We next used Genemania [29] to identify previously known associations between these substrates and the p38 activation pathway we recently reviewed and outlined [30]. Four of the substrates have previously known associations with this pathway (Figure 6C), providing additional support that these targets are relevant and that their phosphorylation could be critical for pathway function.

## Discussion

We have described here a simple technique for kinase substrate finding that uses whole-cell lysate, can identify sites of phosphorylation in addition to the protein phosphorylated, has the ability to identify both *in vivo *and *in vitro *substrates, and can be used to compare the *in vitro *substrate specificity of two kinases in the same experiment. Performing the assay with p38α and p38β revealed that, although differences in preference are apparent, there are no obvious phosphorylation sites specific to p38α. However, a major characteristic that distinguishes p38α from p38β during myoblast differentiation is localisation, with p38α located ubiquitously throughout the cell while p38β is found only at the cell periphery. We also have shown that p38α can phosphorylate several cytosolic phosphorylation sites *in vivo *and that this may be a critical but previously unknown function for it. These results demonstrate the utility of the substrate screening technique we have developed and how its use not only can find proteins phosphorylated by a kinase of interest but also can answer particular questions and propose novel avenues of research.

The technique we have developed has several advantages over existing substrate finding approaches. It can provide direct substrate identifications and information on *in vivo *relevance in a single experiment. Whole-cell lysate can be used as a source of candidate substrates, allowing screens to be performed on samples of specific relevance to the area of interest instead of on protein or peptide arrays. Our approach for eliminating endogenous kinase activity in lysate is nondenaturing, decreasing the likelihood of false-positive and false-negative substrate phosphorylations that would result from the heat-inactivating approach that others have employed [31,32]. At the same time, the recombinant kinase used in the assay does not require mutation or unnatural ATP analogues as other approaches do [33], making the technique applicable to any kinase that can be made and purified in an active form. Although we used dimethyl labelling for quantitative MS, the technique is fully compatible with iTRAQ, which allows up to eight samples to be compared in a single experiment, meaning that simultaneous *in vivo *substrate identification and kinase comparisons can be performed. Radioactivity and gel work are not required, but can be used to visualise phosphorylations and make qualitative comparisons between kinases on one-dimensional gels. The lysate requirements are also relatively low; 1.5 to 3 mg were used in these assays, which may make the technique difficult to employ with some primary cells lines, but it is easily applicable to most secondary cell lines and to tissues as well. We have treated several different lysate types with FSBA, and complete inhibition of endogenous kinase activity occurred in all, meaning that our approach is likely universally applicable. In this study our approach for identifying *in vivo *substrates utilised an inhibitor, but it is more versatile than that, and can be applied to study phosphorylations triggered by a specific stimulus (using stimulated versus unstimulated cells for lysate pools) without requiring a specific inhibitor be available. Finally, although we have performed the *in vivo *approach on unfractionated lysate, when combined with fractionation prior to LC-MS/MS, the number of substrates identified could be much greater than what we have demonstrated in principle here.

As mentioned, a major advantage of the technique presented here is that it can be used to compare the substrate profiles of two or more kinases, and we used this to study the substrate specificity of the p38α and β isoforms. However, we were unable to identify any phosphorylations that were specific to p38α. There are apparent differences in preference for substrates between p38α and p38β, which could be functionally relevant. Assuming that p38β had the same ubiquitous localisation pattern as p38α, a low preference for certain critical myogenic phosphorylations could result in a difference in cell behaviour if p38β were the only isoform present. However, it seems likely that differentiation would still occur, but possibly at a reduced rate or in a compromised way. This is not the case, as p38β-containing myoblasts that lack p38α fail to differentiate at all [7-9,34]. Rather, we believe what truly distinguishes the two isoforms is their localisation. p38α has a known critical role in the nucleus, and our results suggest that the same may be true for the cytoplasm; therefore, p38β would be unable to compensate because it is found solely at the periphery of the cell. A similar distinction exists for two other closely related kinases, focal adhesion kinase (FAK) and proline-rich tyrosine kinase 2 (PYK2) [35]. In fibroblasts, FAK localises to focal adhesions, whereas PYK2 has a perinuclear distribution. PYK2, which has a kinase domain 60% identical to that of FAK, can compensate for the loss of FAK provided it has a focal adhesion targeting sequence. Together these results suggest that rather than substrate specificity, it is other characteristics, in these cases localisation, that distinguish closely related kinases. Whether the same holds true for the entire kinase family is an intriguing question.

In addition to p38α's known role in regulating gene expression at the onset of myoblast differentiation, our results suggest that it is likely to have a cytosolic role as well, possibly in regulating its own pathway. We have found that p38α is present in the cytosol, active p38 increases in the cytoplasm with differentiation, p38α can phosphorylate several cytosolic proteins, three of the cytosolic proteins contain predicted D domains, and connections between these proteins and the p38α activation pathway have been previously reported. Together these results suggest a previously unrecognised role for p38α in the cytosol.

## Conclusions

While the role of the p38α MAPK during myoblast differentiation has been studied extensively with regard to gene regulation, our evidence suggests that this kinase is likely involved in other processes as well. The differentiation of myoblasts into a mature myotube involves extensive morphological and functional changes that p38α might regulate, both through the initiation of a myogenic gene program and through direct phosphorylation and functional modification of cytosolic proteins. No one has previously been able to identify a p38α-specific substrate, and our large-scale screen suggests these may exist only within the context of cellular localisation. In addition, the substrate screening technique we have developed should serve as a useful tool for studying the other kinases known to be involved in myogenesis, as well as the hundreds of other protein kinases that exist.

## Competing interests

The authors declare that they have no competing interests.

## Authors' contributions

JDRK and RK conceived of and designed the project. JDRK conceived of the substrate-finding technique. RT assisted JDRK with the implementation of the substrate-finding technique. RECL performed two-dimensional gel work and assisted JDRK with the development of the radioactive approach. FW assisted with quantitative MS analysis and phosphopeptide validation. AB assisted with cell cultures. All other experiments and analysis were performed by JDRK. JDRK wrote the paper, and RK revised and edited it. HZ supervised FW. LAM supervised RECL and gave advice on project design. DF supervised RT and FW, and DF, TP and ACG provided MS facilities and expertise. RK supervised JDRK. All authors read and approved the final manuscript.

## Supplementary Material

Additional file 1 **Figures S1 through S5 and Tables S1 through S4**. Click here for file
