# Supplementary material for: A novel whole-cell lysate kinase assay identifies substrates of the p38 MAPK in differentiating myoblasts
Source: Skelet Muscle. 2012 Mar 6;2:5. doi: 10.1186/2044-5040-2-5 (PMC3350448; doi:10.1186/2044-5040-2-5)
Supplement: Additional file 1 — Figures S1 through S5 and Tables S1 through S4. [file 2044-5040-2-5-S1.PDF]

Supplemental Material

A novel whole-cell lysate kinase assay identifies substrates of the p38 MAPK in differentiating myoblasts

James D. R. Knight, Ruijun Tian, Robin E. C. Lee, Fangjun Wang, Ariane Beauvais, Hanfa Zou, Lynn A. Megeney, Anne-Claude Gingras, Tony Pawson, Daniel Figeys and Rashmi Kothary

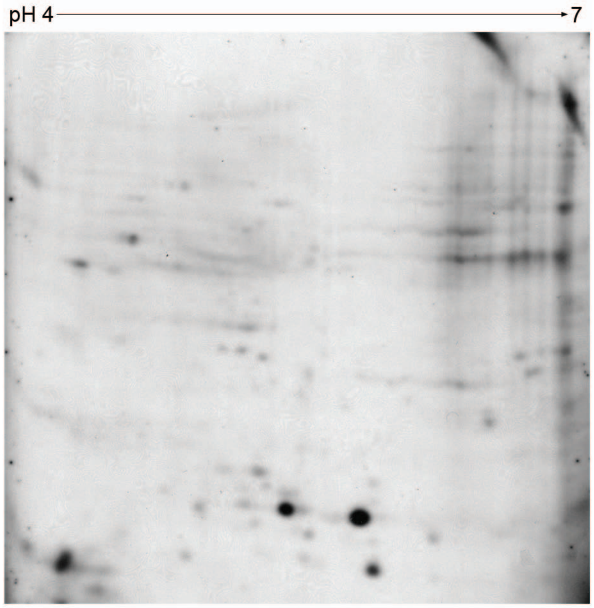

Figure S1 - FSBA is a pan-kinase inhibitor that allows for kinase-specific substrate labeling of cell lysate. After pre-treatment of C2C12 cell lysate with FSBA, purified p38α was added with a kinase assay buffer to specifically label its substrates, visualized via 2D gel electrophoresis.

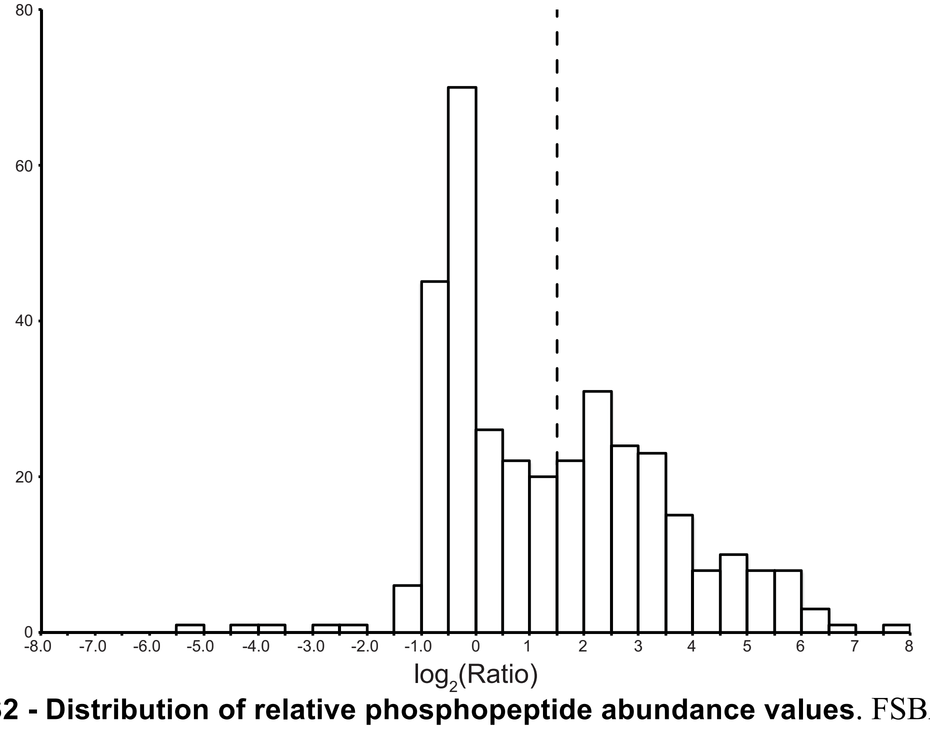

Figure S2 - Distribution of relative phosphopeptide abundance values. FSBA treated lysate was labeled with active p38α (or inactive as a control), followed by dimethyl labeling and a phosphopeptide enrichment as described in the main text. The distribution of the returned abundance ratios (p38α/control) for all phosphopeptides identified is plotted with a bin size of 0.5. The vertical dashed line indicates the fold-cutoff that was used for accepted substrates.

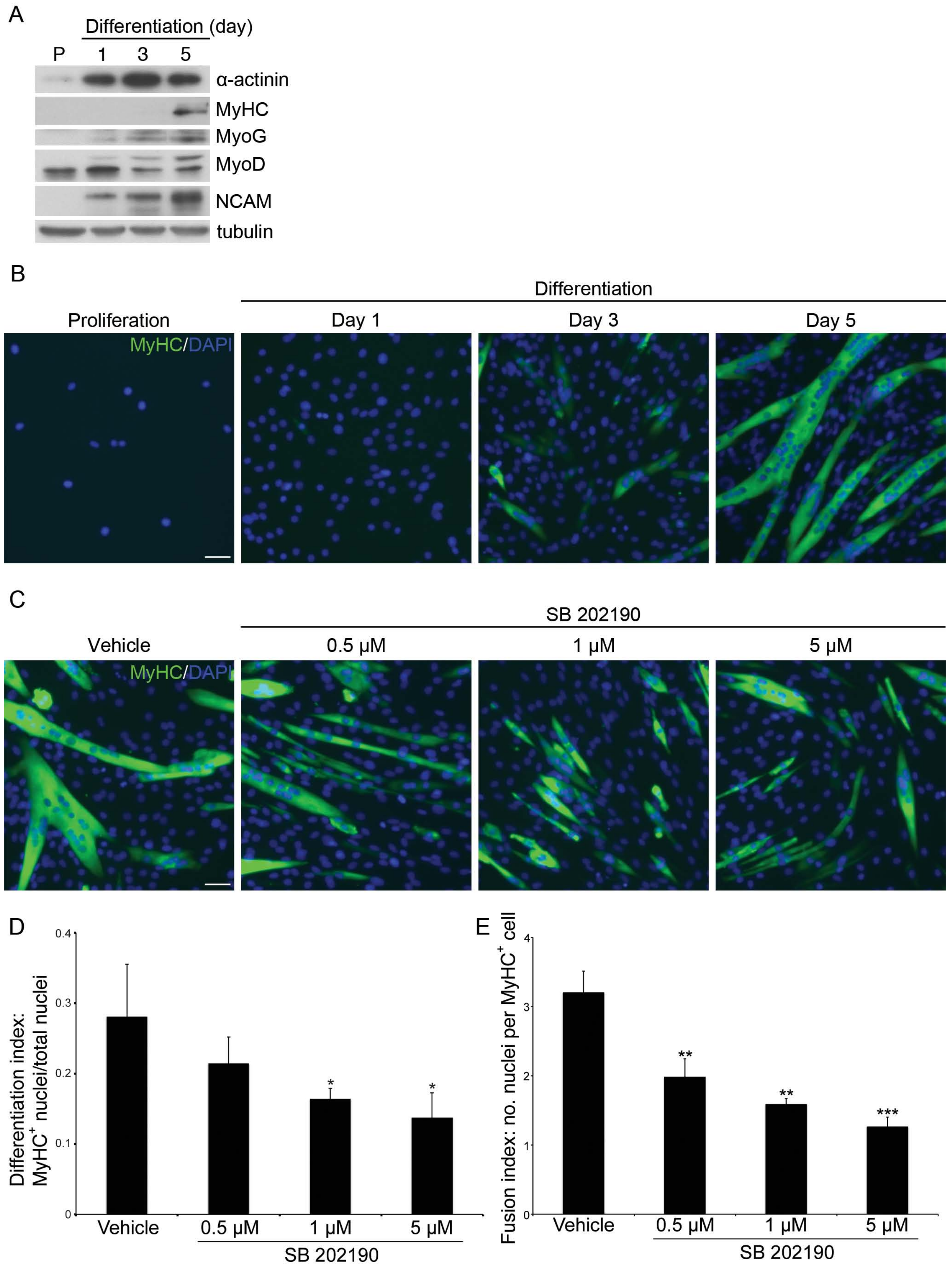
A
Differentiation (day)
P
1
3
5
α-actinin
MyHC
MyoG
MyoD
NCAM
tubulin
B
Differentiation
Proliferation
Day 1
Day 3
Day 5
MyHC/DAPI
C
SB 202190
Vehicle
0.5 μM
1 μM
5 μM
MyHC/DAPI
D
Differentiation index: MyHC+ nuclei/total nuclei
0.4
0.3
0.2
0.1
0
Vehicle
0.5 μM
1 μM
5 μM
SB 202190
E
Fusion index: no. nuclei per MyHC+ cell
4
3
2
1
0
Vehicle
0.5 μM
1 μM
5 μM
SB 202190

Figure S3 - p38 activity is required during the mid to late stages of myoblast differentiation. C2C12 cells were used as a model system for myoblast differentiation. (A) Lysate from proliferating and differentiating C2C12 cells was subjected to western blotting. Differentiating C2C12 cells express typical muscle proteins. (B) Myosin-heavy chain (MyHC) staining of C2C12 cells over a differentiation time course. By day 3 the appearance of multinucleated myotubes is apparent, further augmented by day 5. (C) C2C12 cells were induced to differentiate and at 48 hours the media was supplemented with increasing concentrations of the p38 inhibitor SB202190. On day 5 (120 hours of differentiation), cells were stained for MyHC, a late marker of differentiation. Experiments were performed in triplicate and differentiation and fusion quantified as described in **Methods**. Inhibiting p38 at 48 hours of differentiation causes a reduction in the number of MyHC-positive/differentiated cells (D) and a reduction in myocyte fusion (E). * $p < 0.05$, ** $p < 0.01$, *** $p < 0.001$. Error bars indicate standard deviation. Scale bar = 100 μm.

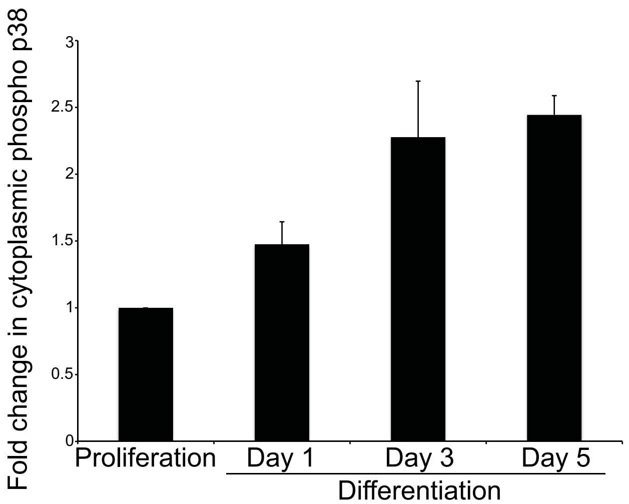

Figure S4 - Quantification of cytoplasmic phospho-p38 levels during C2C12 differentiation from Figure 6A (normalized to tubulin expression). Western blotting was performed in triplicate and the mean is shown with standard deviation. The mean is shown in arbitrary units with proliferation set to 1.

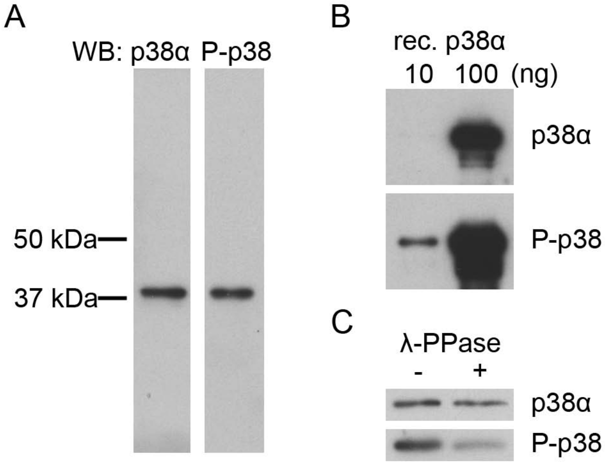

Figure S5 - Validation of the phospho-p38 antibody. (A) Western blotting on C2C12 lysate was performed using p38α and phospho-p38 (P-p38) antibodies. p38α is present as a single band at 38 kDa, therefore all phosphorylated and non-phosphorylated p38α must have the same molecular weight. The phospho-p38 antibody detects a single band at 38 kDa. (B) The phospho-p38 antibody detects recombinant active/phosphorylated p38α (rec. p38α) with an affinity at least as high as the p38α antibody. (C) The phospho-p38 antibody detects p38α with less affinity following λ-phosphatase treatment of purified phosphorylated p38α, while the p38α antibody detects the purified protein with equal affinity regardless of its phosphorylation state.

Table S1 - Phosphopeptides identified using the whole-cell lysate *in vitro* kinase assay with p38α and p38β.

IPI Number	Peptide sequence with phosphorylation sites*	Abundance ratio (p38α/Control)	Abundance ratio (p38β/Control)
IPI00762803	81.03 VELVPPpTPAEIPTAIQSVK b7/y13	21.51676023	22.9665184
IPI00761237	59.35 IILDLISEpSPIKGR b9/y6	1.73435986	1.296315312
IPI00757768	75.38 ITpSPL(ox)MEPSSIEK b3/y11 b6/y8	5.031611919	2.807758331
IPI00134018	41.11 LLPEGEEpTVESDDDKDER b8/y11	0.470338523	0.690812647
IPI00407413	95.15 RAPAAQPPAAAAPSAVGpSPAAAPR b18/y7	31.1769195	34.63834174
IPI00758006	174.39 ATpSNVFA(ox)MFDQSQIQEFK b3/y16 b8/y11	0.714270515	0.597432313
IPI00229201	136.01 GAAQNIIPASpTGAAK b11/y5	9.323296547	4.018772602
IPI00761240	49.23 ENPPpSPHSNSSGK b5/y9	0.731536199	0.801755379
IPI00753321	46.61 EVpSPPGAR b3/y6	0.646028876	0.639157534
IPI00454179	73.55 TNPPTQKPPpSPPVSGR b10/y7	0.654579043	0.628933787
IPI00831454	109.99 TSpSLPGYGK b3/y7	0.719388425	0.602286696
IPI00154054	147.66 FASEIpTPITISVK b6/y8	6.370707989	5.799799919
IPI00911143	65.00 LQPEQGpSPK b7/y3	0.815103352	0.777734995
IPI00911143	99.69 SLpSPLSGTTDTKAESPAGR b3/y17	0.735615318	0.704174133
IPI00116074	35.44 VDVpSPTSQR b4/y6	31.5514164	39.79124832
IPI00473320	81.51 AVFPpSIVGRPR b5/y7	5.03401947	6.817657471
IPI00473320	94.59 EIpTALAPST(ox)MK b3/y9 b10/y2	25.76988983	18.1419754
IPI00473320	113.88 VAPEEHPVLLpTEAPLNPK b11/y8	8.01223433	10.63028932
IPI00221528	98.36 SYELPDGQVIpTIGNER b11/y6	1.652413845	4.627465725
IPI00653007	76.39 VAPEEHPTLLpTEAPLNPK b11/y8	40.64113045	44.2283287
IPI00653007	92.17 VAPEEHPpTLLpTEAPLNPK b8/y11 b11/y8	4.705620766	11.4654274
IPI00856263	89.76 AAVVpTSPPPTTAPHK b5/y11<br>89.76 AAVVTpSPPPTTAPHK b6/y10	1.258880615	0.780669987
IPI00387580	91.57 TEEVLSPDGSPpSKSPSK b12/y6<br>91.57 TEEVLSPDGSPSKpSPSK b14/y4	0.698945701	0.789688528

IPI00553798	64.10 ADIKpTPTVDVTVPEAELNVDSPEINIGGK b5/y25<br>64.10 ADIKTPpTVDVTVPEAELNVDSPEINIGGK b7/y23<br>64.10 ADIKTPTVDVpTVPEAELNVDSPEINIGGK b11/y19	3.218339205	3.755635023
IPI00553798	78.82 ADIKpTPTVDVTVPEAELNVDSPEINIGGK b5/y25<br>78.82 ADIKTPpTVDVTVPEAELNVDSPEINIGGK b7/y23	3.160467148	3.318666697
IPI00553798	119.71 FKAEAPLPpSPK b9/y3	10.11185765	7.639447093
IPI00553798	198.77 AGAIpSASGPELEGAGHSK b5/y14	14.99047661	11.57382107
IPI00553798	99.69 ASLGpSLEGEVEAEASSPK b5/y14	0.746973574	0.636511564
IPI00553798	66.49 ASLGSLEGEVEAEApSSPKGK b15/y6	2.928720713	0.879008293
IPI00553798	75.38 EGVKDIDITpSPEF(ox)MIK b10/y7 b14/y3	2.27114439	5.111543655
IPI00553798	48.83 GGVTGpSPEASISGSKGDLK b6/y14	0.853488323	0.491832657
IPI00553798	61.85 GHYEVpTGSDDEAGKLQGSGVSLASK b6/y20<br>61.85 GHYEVTGpSDDEAGKLQGSGVSLASK b8/y18	0.834961534	0.844889879
IPI00553798	67.76 GKGGVTGpSPEASISGSK b8/y10	1.019585371	0.814918041
IPI00553798	41.92 GKGGVpTGSPEASISGSK b6/y12<br>41.92 GKGGVTGpSPEASISGSK b8/y10	0.979919016	0.795291126
IPI00553798	174.57 GPSFNVApSPESDFGVSLK b8/y11	9.354711056	6.457212687
IPI00553798	28.71 GPSLDIKpSPK b8/y3	7.68009758	6.782256126
IPI00553798	39.82 GPSLKGDLGApSSPS(ox)MK b11/y6 b15/y2<br>39.82 GPSLKGDLGASpSPS(ox)MK b12/y5 b15/y2	2.007005215	0.767946064
IPI00553798	76.39 GSKVDIDpTPQVDVHGPDLK b8/y12	1.995457888	1.63072741
IPI00553798	116.07 IEGSITGPSVEIGpTPDVDVHGLGGK b14/y12	8.106399536	6.444357097
IPI00553798	38.85 IS(ox)MPDIDLHLKpSPK b3/y12 b12/y3	16.38875198	10.54151249
IPI00553798	147.66 LDIDpTPDIDIHGPEGK b5/y12	17.72900613	11.58450969
IPI00553798	78.04 LEGEIKVPDVDIpSSPGINVEAPDIH(ox)MK b13/y15 b26/y2<br>78.04 LEGEIKVPDVDISpSPGINVEAPDIH(ox)MK b14/y14 b26/y2	6.015406132	3.353409767
IPI00553798	128.31 LEGEIKVPDVDISpSPGINVEAPDIH(ox)MK b14/y14 b26/y2	2.840684891	1.673624158
IPI00553798	64.21 LPQFGIpSTPGSDLDINIK b7/y12<br>64.21 LPQFGISpTPGSDLDINIK b8/y11	6.302074432	8.361244202
IPI00553798	91.91 LPQFGISpTPGSDLDINIK b8/y11	3.952844381	5.034689426
IPI00553798	28.02 LPSGSGPApSPTTGSAVDIR b9/y11	1.514978886	1.391719699

IPI00553798	28.02 LPpSGSGPASPTTGSAVDIR b3/y17<br>28.02 LPSGpSGPASPTTGSAVDIR b5/y15<br>28.02 LPSGSGPApSPTTGSAVDIR b9/y11	1.494694233	1.499192357
IPI00553798	42.72 LPpSGSGPApSPTTGSAVDIR b3/y17 b9/y11<br>42.72 LPSGpSGPApSPTTGSAVDIR b5/y15 b9/y11	0.939601362	0.992946565
IPI00553798	71.80 LRpSEDGVEGDLGETQSR b3/y15	0.563569069	0.600886464
IPI00553798	57.59 (ox)MPFLSIpSSPK b1/y10 b7/y4<br>57.59 (ox)MPFLSISpSPK b1/y10 b8/y3	203.2595215	112.4314575
IPI00553798	81.51 (ox)MPFLSISpSPK b1/y10 b8/y3	66.5812912	44.76893234
IPI00553798	96.66 SNSFSDEREFSAPpSTPTGTLEFAGGDAK b14/y15<br>96.66 SNSFSDEREFSAPSpTPTGTLEFAGGDAK b15/y14	4.731276989	4.313008785
IPI00553798	49.83 pSNSFSDEREFSAPpSTPTGTLEFAGGDAK b1/y28 b14/y15<br>49.83 SNpSFSDEREFSAPpSTPTGTLEFAGGDAK b3/y26 b14/y15<br>49.83 SNSFpSDEREFSAPpSTPTGTLEFAGGDAK b5/y24 b14/y15<br>49.83 pSNSFSDEREFSAPSpTPTGTLEFAGGDAK b1/y28 b15/y14<br>49.83 SNpSFSDEREFSAPSpTPTGTLEFAGGDAK b3/y26 b15/y14<br>49.83 SNSFpSDEREFSAPSpTPTGTLEFAGGDAK b5/y24 b15/y14	4.523502827	5.059371948
IPI00553798	71.69 SNpSFSDEREFSAPSTPTGTLEFAGGDAK b3/y26<br>71.69 SNSFpSDEREFSAPSTPTGTLEFAGGDAK b5/y24	0.055511478	0.246567145
IPI00553798	96.60 pSNSFSDEREFSAPSTPTGTLEFAGGDAK b1/y28<br>96.60 SNpSFSDEREFSAPSTPTGTLEFAGGDAK b3/y26	0.029090421	0.01622114
IPI00553798	133.43 SSEVVLpSGDDEDYQR b7/y9	0.697577119	0.696674883
IPI00553798	75.25 TVIRLPSGSGPApSPTTGSAVDIR b13/y11<br>75.25 TVIRLPSGSGPASPpTTGSAVDIR b15/y9	1.445720315	0.668393075
IPI00553798	84.91 TVIRLPpSGSGPApSPTTGSAVDIR b7/y17 b13/y11<br>84.91 TVIRLPSGpSGPApSPTTGSAVDIR b9/y15 b13/y11	0.854798615	0.433122545
IPI00553798	82.80 TVIRLPSGpSGPApSPTTGSAVDIR b9/y15 b13/y11	0.809538841	0.524245262
IPI00553798	106.28 TVIRLPpSGSGPASPTTGSAVDIR b7/y17<br>106.28 TVIRLPSGpSGPASPTTGSAVDIR b9/y15	0.07723733	0.108180381
IPI00553798	164.13 VDIDpTPQVDVHGPDLK b5/y12	5.659213543	5.218640804
IPI00553798	65.70 VDLEpTPSLDVH(ox)MESPDINIEGPDVK b5/y21 b12/y14<br>65.70 VDLETPpSLDVH(ox)MESPDINIEGPDVK b7/y19 b12/y14	5.35586977	4.504248857

IPI00553798	76.28 VDLEpTPSLDVH(ox)MEpSPDINIEGPDVK b5/y21 b12/y14 b14/y12	3.907617807	1.853503942
IPI00553798	107.40 VDLETPSLDVH(ox)MEpSPDINIEGPDVK b12/y14 b14/y12	1.839476824	3.798395872
IPI00553798	56.79 VDLEpTPSLDVH(ox)MESPDINIEGPDVK b5/y21 b12/y14	1.809990406	2.280708075
IPI00553798	138.22 VPDVDIpSSPGINVEAPDIH(ox)MK b7/y15 b20/y2	9.175672531	7.833360672
IPI00553798	124.10 VPDVDIpSSPGINVEAPDIH(ox)MK b7/y15 b20/y2<br>124.10 VPDVDISpSPGINVEAPDIH(ox)MK b8/y14 b20/y2	2.940662821	2.841840148
IPI00553798	106.41 VQpTPEVDVK b3/y7	56.81110764	48.3420639
IPI00553798	114.49 VSVApTPDVSLEASEGAVK b5/y14	5.698278785	3.542760086
IPI00605894	76.28 ADIDVpSGPKVDIDVPDVNIEGPDAK b6/y20	0.91219002	0.933866918
IPI00605894	89.76 AEpSPE(ox)MEVNLPK b3/y10 b6/y7	0.948591053	1.510591865
IPI00605894	82.80 ATIDVSGPKLDIEpTSDVSLEGPEGK b14/y12<br>82.80 ATIDVSGPKLDIETpSDVSLEGPEGK b15/y11	6.461926937	4.198677063
IPI00605894	106.25 GPDINLPEVpSVKTPK b10/y6	21.87118721	23.05458641
IPI00605894	40.26 GSRVDIEpTPNLEGTLTGPK b8/y12,	6.218453884	1.737978339
IPI00605894	40.22 IpSSPSGK b2/y6, 40.22 ISpSPSGK b3/y5	1.154801369	1.851094961
IPI00856247	109.95 GILAADESpTGSIAKR b9/y7	29.69270134	15.15349483
IPI00172197	21.90 pSCTKPSPSK b1/y9	0.939577937	0.804510415
IPI00230395	87.24 GGPGpSAVpSPYPSFNVSSDVAALHK b5/y20 b8/y17	3.443393469	2.932432175
IPI00230395	91.57 GGPGpSAVSPpYPSFNVSSDVAALHK b5/y20 b10/y15	3.356889963	2.932432175
IPI00230395	87.76 GGPGpSAVSPYPSFNVSSDVAALHK b5/y20	2.964440227	3.096542358
IPI00310240	83.62 DQAQEDAQEIADpTPSGDKTSLETR b13/y12	3.476381302	3.741753817
IPI00754418	60.79 TSEDTSSGpSPPKK b9/y5	0.68289423	0.894969821
IPI00754418	83.47 TSEDpTSSGSPPKKSPGGPK b5/y15<br>83.47 TSEDTpSSGSPPKKSPGGPK b6/y14<br>83.47 TSEDTSpSGSPPKKSPGGPK b7/y13<br>83.47 TSEDTSSGpSPPKKSPGGPK b9/y11	0.814678729	0.614110351
IPI00170307	139.42 RGpSET(ox)MAGAAVK b3/y10 b6/y7	0.682991385	0.723003387
IPI00224070	65.55 SSpSPEPVTHLK b3/y9	2.056138992	1.987481236
IPI00224070	83.81 SpSSPEPVTHLK b2/y10<br>83.81 SSpSPEPVTHLK b3/y9	1.854281425	2.027197361

IPI00322312	161.03 AEEYEFLpTP(ox)MEEAPK b8/y8 b10/y6	8.238176346	6.729856014
IPI00468481	136.01 VLDpSGAPIKIPVGPETLGR b4/y16	24.97766495	24.40690041
IPI00169500	81.51 STSTPTpSPGPR b7/y5	10.68512535	9.208967209
IPI00129519	81.42 AEGAGTEEEGpTPKESEPQAAADATEVK b11/y17	1.029820204	2.010046482
IPI00129519	119.94 SDAAPAASDSKPpSSAEPAPSSK b13/y10<br>119.94 SDAAPAASDSKPSpSAEPAPSSK b14/y9	1.590026975	7.439866543
IPI00415385	81.51 EVQpSPEQVKSEK b4/y9	0.634465396	0.453926951
IPI00415385	91.73 IDISPpSALR b6/y4	0.6715042	0.661963761
IPI00282748	130.95 GNKpSPSPPPDGSPAATPEIR b4/y17	0.822660387	0.850421548
IPI00282748	105.78 GNKSPpSPPPDGSPAATPEIR b6/y15	0.57112509	0.61547929
IPI00282748	73.29 VNHEPEPASGApSPGATIPK b12/y8	0.553376339	0.761530601
IPI00311344	89.63 GSVFSAPpSASGTPNKETAGLK b8/y14<br>89.63 GSVFSAPSApSGTPNKETAGLK b10/y12<br>89.63 GSVFSAPSASGpTPNKETAGLK b12/y10	15.6720953	17.13145828
IPI00311344	101.78 GSVFSAPpSASGTPNKETAGLK b8/y14<br>101.78 GSVFSAPSApSGTPNKETAGLK b10/y12	13.53505516	11.50659084
IPI00311344	77.31 QSVDKVpTSPTKV b7/y6<br>77.31 QSVDKVTpSPTKV b8/y5	1.175395727	0.932990015
IPI00399958	62.30 SFDQLpTPEESKER b6/y8	0.591723025	1.443731308
IPI00119618	73.55 AEEDEILNRpSPR b10/y3	0.758565954	0.909349354
IPI00119618	180.59 APVPpTGEVYFADSFDR b5/y12	4.525886536	4.148888588
IPI00133349	65.55 GNVVPpSPLPTRR b6/y7	0.868576044	0.739671483
IPI00133349	63.74 TFpSATVR b3/y5	0.763473213	0.760139287
IPI00230645	130.88 LSAAISEVVSQpTPAPSTHAAAPLPGTEQK b12/y18	3.589641571	1.996758342
IPI00230645	35.73 VTASSAApTSKSPS(ox)MSTTETK b8/y13 b14/y7<br>35.73 VTASSAATpSKSPS(ox)MSTTETK b9/y12 b14/y7<br>35.73 VTASSAATSKpSPS(ox)MSTTETK b11/y10 b14/y7	0.636452913	0.667064607
IPI00381495	73.55 LDQPVSAPPpSPR b10/y3	1.189889908	1.506361842
IPI00753875	154.47 SLpSADNFIGIQR b3/y10	0.721474349	0.772702277
IPI00653274	124.10 LALVpTGGEIASTFDHPELVK b5/y16	7.553201675	10.19968319

IPI00113849	28.71 YVECpSALTQK b5/y6	1.808095932	1.343613386
IPI00227808	54.09 TAPVQAPPAPVTVTEpTPEPA(ox)MPSGVYRPPGAR b16/y17 b21/y12	2.251980066	4.621519566
IPI00136703	150.12 VLpTPELYAELR b3/y9	9.626471519	8.553112984
IPI00828500	122.72 EQTASAPApTPLVSK b9/y6	6.829474449	6.444790363
IPI00828500	69.06 ITVEKDPDSALGIpSDGETSPSSK b14/y10	0.748685696	0.699242297
IPI00828500	48.06 STpSVDDTDKSSSEAI(ox)MVR b3/y16 b16/y3	0.623449147	0.632381797
IPI00109588	118.15 GFPGPPGPDGLPGS(ox)MGPPGpTPSVDHGFLVTR b15/y17 b20/y12	1.560830474	2.374943972
IPI00123891	202.39 GFGFGQGAGALVHpSE b14/y2	0.770651281	0.768593609
IPI00468516	78.04 VDNARVpSPEVGSADVASIAQK b7/y15	0.537959574	0.474731256
IPI00775841	56.74 LpSSPVLHR b2/y7<br>56.74 LSpSPVLHR b3/y6	1.061162829	0.739433467
IPI00458127	106.25 SAGAPRpTGEPEQEAVSR b7/y11	1.522797346	1.508806348
IPI00881493	37.98 SPSPSPpTSPGSLRK b7/y8<br>37.98 SPSPSPTpSPGSLRK b8/y7	0.529263377	0.48207593
IPI00130102	162.62 TFGGAPGFSLGSPLpSSPVFPR b15/y7<br>162.62 TFGGAPGFSLGSPLSpSPVFPR b16/y6	5.576372385	5.621459842
IPI00130102	123.02 TFGGAPGFSLGSPLSpSPVFPR b16/y6	6.713711262	5.781813383
IPI00130102	110.44 TFGGAPGFSLGpSPLSpSPVFPR b12/y10 b16/y6	2.479888678	3.487055063
IPI00130102	138.17 TFGGAPGFSLGpSPLSpSPVFPR b12/y10 b16/y6<br>138.17 TFGGAPGFSLGSPLpSpSPVFPR b15/y7 b16/y6	2.067015171	2.880161524
IPI00134809	52.96 AKPAEpTPAPAHK b6/y7	24.51044846	23.23945236
IPI00894804	147.40 SLSTpSGESLYHVLGLDK b5/y13	0.546143889	1.006157994
IPI00114375	61.05 pTSPAKQQAPPVR b1/y12<br>61.05 TpSPAKQQAPPVR b2/y11	0.526955187	0.374703526
IPI00122349	57.59 GSPpTRPNPPVR b4/y8	0.763181925	0.817926824
IPI00153421	95.78 SVSSNVASVpSPIPAGSKK b10/y9	11.63234901	8.854105949
IPI00330066	130.88 TRpSPDVISSASTALSQDIPEIASEALSR b3/y26	0.680407107	0.739012182
IPI00307837	99.63 VEpTGVLKPG(ox)MVVTFAPVNVTTEVK b3/y22 b10/y15	7.897317444	6.000155551
IPI00307837	90.42 VEpTGVLKPG(ox)MVVTFAPVNVpTTEVK b3/y22 b10/y15 b20/y5<br>90.42 VEpTGVLKPG(ox)MVVTFAPVNVTpTEVK b3/y22 b10/y15 b21/y4	5.384685516	4.658336163

IPI00307837	101.22 VEpTGVLKPG(ox)MVVTFAPVNVpTTEVK b3/y22 b10/y15 b20/y5	4.681442738	6.01963377
IPI00620302	112.44 GATPAEDDEDKDIDLFGpSDEEEEDKEAAR b18/y12	0.635066763	0.563856326
IPI00115992	106.41 LIpTPAVVSER b3/y8	11.07410049	9.419182777
IPI00620806	83.38 SEpSPKEPEQLR b3/y9	0.982602655	1.160811961
IPI00409405	56.67 LGGpSAVISLEGKPL b4/y11	15.88397408	14.62335873
IPI00321647	98.01 QPLLLpSEDEEDTKR b6/y9	0.70149942	0.686574039
IPI00421179	101.82 AApSLTEDR b3/y6	0.870357871	1.274669528
IPI00421179	69.17 EATLPPVpSPPK b8/y4	0.843857368	0.732594743
IPI00648821	111.19 EALELLKpTAIAK b8/y5	11.89870739	11.00475693
IPI00648821	174.30 YIpTPDQLADLYK b3/y10	0.869313419	1.290469646
IPI00122684	191.05 AAVPSGApSTGIYEALELR b8/y11<br>191.05 AAVPSGASpTGIYEALELR b9/y10	35.18431346	37.44134808
IPI00622968	144.53 FAAATGApTPIAGR b8/y6	27.76387215	23.59006119
IPI00173160	119.47 LVQpSPNSYF(ox)MDVK b4/y10 b10/y4	3.612674475	1.551651597
IPI00420187	179.98 TVFAGAVPVLPApSPPPKDSLR b13/y9	0.913019776	0.814337611
IPI00116753	76.62 LNVAPVSDIIEIKpSPDTFVR b14/y7	6.699317932	5.57572031
IPI00154109	100.39 HGLLLPApSPVR b8/y4	1.859685421	2.273117542
IPI00113223	91.57 LFDHPEVPpTPPESApSVSR b9/y10 b15/y4	5.942027569	6.022670269
IPI00113223	103.96 LFDHPEVPTPPEpSASVSR b13/y6<br>103.96 LFDHPEVPTPPESApSVSR b15/y4	2.781153917	4.825872898
IPI00875567	61.85 FGGEHVPNSPFQVTALAGDQPTVQpTPLR b25/y4	8.982500553	8.96617047
IPI00875567	67.76 VATVPQHApTSGPGPADVSK b9/y11	15.74446535	8.582057953
IPI00663627	125.18 VLFASQEIPApSPFR b11/y4	17.37866211	14.38440704
IPI00664670	65.00 LGpSFGSITR b3/y7	0.723294854	0.886812866
IPI00331295	102.85 SApTPPPAEPASLPQEPPKPR b3/y18	0.722537458	0.821812153
IPI00111960	86.47 TpSPTFFPK b2/y7	8.900146484	15.0602684
IPI00111960	91.73 YEVPLEpTPR b7/y3	10.62809086	14.5218792
IPI00108811	174.39 NFVDpSPIIVDIPK b5/y9	4.659234524	3.191150188
IPI00165716	124.10 ADASSLTVDVpTSPASKVPTTVEDR b11/y14	4.119154453	3.174692631

	124.10 ADASSLTVDVTpSPASKVPTTVEDR b12/y13		
IPI00228633	70.15 ELQAAGKpSPEDLEK b8/y7	33.58008194	38.31991959
IPI00759948	77.31 AALKpTASDFISK b5/y8	35.98693085	29.98003197
IPI00759948	67.31 DGGQpTAPASIR b5/y7	71.81306458	39.77776718
IPI00153986	91.57 GTSRPGpTPSAEAASTSSTLR b7/y14<br>91.57 GTSRPGTPpSAEAASTSSTLR b9/y12	5.166966438	6.633099556
IPI00828543	71.39 TIpTLVKSPISVPGGSALISNLGK b3/y21<br>71.39 TITLVKpSPISVPGGSALISNLGK b7/y17	5.665997982	3.815047979
IPI00882349	113.87 AGDVLEDpSPKRPK b8/y6	0.799336821	0.767371282
IPI00882349	65.28 GSAEGpSpSDEEGKLVIDEPAKEK b6/y17 b7/y16	0.958018771	1.259046912
IPI00331556	100.39 VLApTAFDTTLGGR b4/y10	1.710803151	1.556209803
IPI00319992	167.38 ITPSYVAFpTPEGER b9/y6	17.79181099	15.56201363
IPI00323357	75.25 IINEPpTAAAIAYGLDKK b6/y12	33.94918823	16.67408371
IPI00133903	167.38 TpTPSVVAFTADGER b2/y13	7.578380108	11.57881355
IPI00468068	44.84 AQIGGPEAGKpSEQSGAK b11/y7	58.96623993	107.8104477
IPI00468068	53.05 AQIGGPEAGKSEQpSGAK b14/y4	5.323335171	12.02662373
IPI00468068	138.72 AVTQpSAEITIPVTFEAR b5/y13	48.22555161	68.71230316
IPI00468068	111.42 QLpSSGVSEIR b3/y8	0.723811746	0.684148848
IPI00224109	86.47 IEpSPKLER b3/y6	0.748215318	0.636383057
IPI00308971	78.04 AEALSSLHGDDQDpSEDEVLTVPEVK b14/y12	0.768615842	0.734100819
IPI00319956	90.47 TEEVEVEpSEEDPILEHPPENPVK b8/y16	0.525107026	0.501573026
IPI00889930	84.26 AGGApSPAASSTTQPPAQHR b5/y15	0.786503077	0.675689697
IPI00856218	154.78 AApSALLLR b3/y6	0.723594725	0.692247629
IPI00856218	66.57 ELpSAPAR b3/y5	0.704663932	0.641427338
IPI00331173	77.31 SVpSASHEGDVK b3/y9<br>77.31 SVSApSHEGDVK b5/y7	0.723999858	0.634566069
IPI00751369	100.07 VTLpTPEEEAR b4/y7	81.00878143	65.96704102
IPI00112339	62.96 ELpSVEEQIKR b3/y8	1.014968038	1.0225389
IPI00112339	83.38 TSSIKpSPK b6/y3	2.33336401	2.328483582

IPI00400300	67.31 LRLpSPpSPTSQR b4/y8 b6/y6<br>67.31 LRLSPpSPpTSQR b6/y6 b8/y4	0.811540782	0.797581792
IPI00400300	88.90 LSPpSPTSQR b4/y6	0.7242257	0.744147837
IPI00230394	85.46 ASAPATPLpSPTR b9/y4	4.817977428	3.001209259
IPI00659447	83.81 LAPpSPSEEPR b4/y7	0.614996552	0.736216784
IPI00659447	65.00 TSPGpSPSPR b5/y5	4.849502087	6.031260967
IPI00757916	81.51 AGDLGVDLpTSK b9/y3	12.79569435	12.37896442
IPI00757916	37.98 LPAVVpTADLR b6/y5	9.101158142	6.789206505
IPI00849751	83.81 IEEELGpSKAK b7/y4	12.49800158	15.97111464
IPI00849165	105.56 SApSPGLPKGEK b3/y9	0.706182406	0.570384
IPI00606906	131.17 AVLPGpSPIFSR b6/y6	9.220336795	9.256473896
IPI00785324	48.83 AENQRPAEDSALpSPGPLAGAK b13/y9	1.23474443	0.904589415
IPI00319270	206.91 TLTTAAVpSTAQPILSK b8/y9<br>206.91 TLTTAAVSpTAQPILSK b9/y8	1.352540374	1.087141275
IPI00830432	99.69 QGQDVAPPPNPVPQRpTSPTGPK b16/y7<br>99.69 QGQDVAPPPNPVPQRTpSPTGPK b17/y6	0.690261126	0.499007702
IPI00229534	112.17 AEDGAAPpSPSSETPK b8/y8	2.100413918	3.392316341
IPI00229534	92.17 AEDGAAPSPpSSETPKK b10/y7<br>92.17 AEDGAAPSPSpSETPKK b11/y6	9.322688103	3.320444107
IPI00229534	91.91 AEDGAAPpSPSSETPKKK b8/y10<br>91.91 AEDGAAPSPpSSETPKKK b10/y8<br>91.91 AEDGAAPSPSpSETPKKK b11/y7<br>91.91 AEDGAAPSPSSEpTPKKK b13/y5	10.36584091	3.589919329
IPI00229534	74.81 AEDGAAPpSPSSEpTPKKK b8/y10 b13/y5	5.159576893	3.845372438
IPI00229534	103.82 GEATAERPGEAAVApSSPSK b15/y5<br>103.82 GEATAERPGEAAVASpSPSK b16/y4	0.772615953	1.101140915
IPI00323820	45.48 ISDPLpTSSPGR b6/y6<br>45.48 ISDPLTpSSPGR b7/y5<br>45.48 ISDPLTSpSPGR b8/y4	0.456634462	0.542350292
IPI00323592	119.47 NLGIGKIpTPFEEK b8/y6	16.57404327	9.897894859
IPI00323592	156.23 VAVLGASGGIGQPLpSLLLKNSPLVSR b15/y12	5.287952423	4.215777397

IPI00323592	130.17 VAVLGASGGIGQPLSLLLKNpSPLVSR b21/y6	5.2212677	3.951768398
IPI00315808	93.44 GSTpSPDLL(ox)MHQGPPDTAEIIK b4/y18 b9/y13	3.265588999	1.351506114
IPI00408909	89.76 ALALVPGpTPTR b8/y4	7.481154442	5.125487804
IPI00896700	35.34 ETAAAHQApSSSPPIDAATAEPYGFR b9/y17<br>35.34 ETAAAHQASpSSPPIDAATAEPYGFR b10/y16<br>35.34 ETAAAHQASSpSPPIDAATAEPYGFR b11/y15	0.662014365	0.767188072
IPI00896700	103.12 RSEpSPFEGK b4/y6	0.78600955	0.6921826
IPI00896700	91.73 SDIpSPLTPR b4/y6	0.249109641	0.321611315
IPI00896700	78.04 SPSLSPSPPpSPIEK b10/y5	0.783955634	0.786213338
IPI00896700	20.59 SVpSPGVTQAVVEEHCASPEEK b3/y19	0.747820914	0.549286246
IPI00896700	72.16 TTpTTPEVK b3/y6<br>72.16 TTTpTPEVK b4/y5	4.88103199	4.319426537
IPI00896700	81.42 VLpSPLRSPPLLGSESPYEDFLSADSK b3/y24	1.197787881	0.923976958
IPI00896700	91.91 VLpSPLRpSPPLLGSESPYEDFLSADSK b3/y24 b7/y20	1.015276074	1.005762339
IPI00896700	93.74 VLpSPLRpSPPLLGSESPYEDFLSADSK b3/y24 b7/y20<br>93.74 VLpSPLRSPPLLGpSESPYEDFLSADSK b3/y24 b13/y14	1.005020618	1.075676441
IPI00896700	71.39 VLpSPLRSPPLLGSESPpYEDFLSADSK b3/y24 b17/y10<br>71.39 VLSPLRpSPPLLGSESPpYEDFLSADSK b7/y20 b17/y10	0.907762647	0.704117596
IPI00896700	109.03 VLpSPLRpSPPLLGSESPYEDFLSADSK b3/y24 b7/y20	0.859545767	0.744838953
IPI00896700	154.48 VLSPLRpSPPLLGSESPYEDFLSADSK b7/y20	0.641937673	0.583019137
IPI00408119	78.04 AAVGVTGNDITTPPNKEPPPpSPEKK b21/y5	1.094480515	0.926650345
IPI00408119	140.55 ALET(ox)MAEQTTDVVHpSPSTDTTPGPDTEAALAK b5/y28 b15/y18	1.170022845	0.966159225
IPI00408119	70.19 D(ox)MpSPLPESEVTLGKDVVILPETK b2/y22 b3/y21	2.095468521	1.406081438
IPI00310519	150.12 AELAHpSPLPAK b6/y6	49.89553452	20.81400681
IPI00123199	111.87 LDGLVDpTPTGYIESLPK b7/y11<br>111.87 LDGLVDTPpTGYIESLPK b9/y9	10.2134161	14.16672611
IPI00123199	171.74 LDGLVDpTPTGYIESLPK b7/y11	4.824375153	5.192005157
IPI00845608	158.15 APLVGpSPVHLGPSQPLK b6/y12	1.130340576	1.063784406
IPI00845608	98.36 ARpTPTLASpTPIPP(ox)MSEAPYPK b3/y19 b9/y13 b14/y8	6.97160244	2.749439478
IPI00845608	121.98 ESQEFLRpSPEAEEEEEQV(ox)MVR b8/y14 b19/y3	1.00393486	1.102925181

IPI00845608	68.56 QEpSLKSPEEEDQQAFR b3/y14<br>68.56 QESLKpSPEEEDQQAFR b6/y11	1.111553073	1.09843564
IPI00845608	46.94 VSQVSLESLEKENVQpSPR b16/y3	2.127660751	1.682767034
IPI00400168	67.05 AEIKE(ox)MLApSDDEEESSPK b6/y13 b9/y10	0.408290505	1.04345119
IPI00127417	67.05 V(ox)MLGETNPADpSKPGTIR b2/y16 b11/y7	0.636349976	1.54293561
IPI00341869	89.76 ATVTPpSPVKGK b6/y6	0.548258601	0.37350271
IPI00469331	62.87 GVLSpSPSLAFTTPIR b5/y11	2.772338629	3.383033514
IPI00126313	48.20 PLHYLpTILpSPR b6/y6 b9/y3	9.889798641	6.283292055
IPI00282266	63.74 EIQpTAVR b4/y4	11.28819752	17.82034302
IPI00624863	119.71 EVYELLDpTPGR b8/y4	12.44848633	18.17038918
IPI00229884	97.24 IIpSIFSGTEK b3/y8	0.746232986	0.614436567
IPI00762775	81.51 SVPGVTpSTPHSK b7/y6<br>81.51 SVPGVTSpTPHSK b8/y5<br>81.51 SVPGVTSTPHpSK b11/y2	29.33709908	15.29289818
IPI00762775	130.07 TLpS(ox)MIEEEIR b3/y8 b4/y7	0.489557877	0.452896486
IPI00470003	52.96 SPLVPKpSPTPK b7/y5	2.232306719	1.605970263
IPI00470003	99.69 SPLVPKpSPTPKpSPPSR b7/y10 b12/y5	1.364190578	1.398820877
IPI00470003	71.15 SPTPKpSPPSR b6/y5	0.139414813	0.046658279
IPI00453818	73.45 EPSAPSIPPPAYQSSPAAGHAAAPPpTPAPR b26/y5	4.204166889	4.657279968
IPI00337893	30.65 YG(ox)MGpTSVER b3/y7 b5/y5<br>30.65 YG(ox)MGTpSVER b3/y7 b6/y4	0.642371833	0.643605232
IPI00309768	116.92 SA(ox)MPFpTASPAPSTR b3/y12 b6/y9	41.06153107	38.69659424
IPI00153375	214.41 FSpSLDLEEDSEVFK b3/y12	0.587694585	0.585939825
IPI00828969	100.39 EVVKPVPITpSPAVSK b10/y6	46.95588112	29.39895439
IPI00405307	61.05 ALpTPPADPPR b3/y8	1.889647484	1.495816231
IPI00336400	100.39 DIIRQPpSEEEIIK b7/y7	0.512478398	0.587568969
IPI00336400	91.73 QPpSEEEIIK b3/y7	0.511900408	0.555279012
IPI00387312	72.16 TLpSIDKGF b3/y6	0.661338091	0.634651005
IPI00555069	111.19 AHpSS(ox)MVGVNLPQK b3/y11 b5/y9<br>111.19 AHSpS(ox)MVGVNLPQK b4/y10 b5/y9	2.046526432	0.741505384

IPI00555069	90.47 ALEpSPERPFLAILGGAK b4/y14	5.652894735	7.445711851
IPI00132080	62.30 IVAPISDpSPKPPPQR b8/y8	48.8340683	45.37990189
IPI00319973	107.82 EGEEPTVYpSDDEEPKDETAR b9/y12	1.366412997	1.955558419
IPI00319973	109.03 LLKEGEEPpTVYSDDEEPKDETAR b9/y15<br>109.03 LLKEGEEPTVYpSDDEEPKDETAR b12/y12	0.945469558	0.735155523
IPI00319973	86.23 LLKEGEEPTVYpSDDEEPKDETAR b12/y12	0.785713136	0.698052168
IPI00400381	122.16 SSpSPVLVEEEPER b3/y11	0.70067066	0.660208404
IPI00407130	100.11 EApTESFASDPILYRPVAVALDTK b3/y21	1.235897183	2.331736326
IPI00407130	102.90 LDIDSAPIpTAR b9/y3	12.86094761	15.59814262
IPI00626385	81.03 EREEGAPEpTPVVSATTVGTLAR b9/y14	3.078224182	2.917778015
IPI00120546	57.59 ALDDFVLGpSAR b9/y3	1.821764469	1.868702412
IPI00831115	72.63 SAPDFpTATAVVDGAFKEIK b6/y14<br>72.63 SAPDFTApTAVVDGAFKEIK b8/y12	1.674755454	2.067908049
IPI00875652	127.75 TDSREDEIpSPPPPNPVVK b9/y10	0.72280697	0.645957748
IPI00126939	58.08 GESALEPGPVPEpTPAGGPVHAVTVVTLLEK b13/y18	0.668460131	0.959567428
IPI00875405	130.95 TASAVAGKpTPDASPEPK b9/y9	5.994947195	7.970684767
IPI00381291	185.98 AAAASAAEAGIApTPGTEGERDSDDALLK b13/y16	4.361035824	4.936915278
IPI00759871	68.55 IITGPAPVLPPAALRpTPTPAGPTI(ox)MPLIR b16/y14 b25/y5	3.563090324	4.916073799
IPI00830159	51.81 AGQGIPAPPEASPTAVPEPpSTPFPPVLASG(ox)MSHPPPTSR b20/y20 b31/y9	2.539334059	12.50415802
IPI00828741	61.05 GRLpSPVPVPR b4/y7	1.472668052	1.775638342
IPI00135190	126.36 TSpSLTHSEEK b3/y8<br>126.36 TSSLpTHSEEK b5/y6	0.521138012	1.325824022
IPI00649362	54.78 QPpTPPFFGR b3/y7	0.816814005	0.618681371
IPI00124826	149.91 LEPAPLDpSSPAVSTHEGSK b8/y12<br>149.91 LEPAPLDSpSPAVSTHEGSK b9/y11	4.544881105	4.156165838
IPI00133185	91.73 AAILKApSPK b7/y3	3.479880571	4.195896149
IPI00742383	16.12 LASVPAGGAVAVpSAAPGSAAPAAGSAPAAAEEKKDEK b13/y25	4.492766857	3.956872225
IPI00742383	78.04 YVASYLLAALGGNSpSPSAK b15/y5	1.471876264	0.945997953
IPI00762542	126.36 NIGLGFKpTPK b8/y3	29.15384865	25.75343132

IPI00323819	139.42 LIDLHpSPSEIVK b6/y7	9.320585251	11.50042343
IPI00469392	87.23 RGSGSVDEpTLFALPAASEPVIPSSAEK b9/y19	0.895802438	1.101232767
IPI00122174	49.83 APTAALpSPEPQDSKEDVK b7/y12	1.04063201	0.9422822
IPI00114733	56.79 DQAVENILLpSPLVVASSLGLVSLGGK b10/y17	5.865635395	2.852160454
IPI00895328	54.78 RPTEAVpSPK b7/y3	0.713203669	0.70842886
IPI00116331	101.93 APDRpTPPSEEDSAEAER b5/y13	0.847935557	0.838952303
IPI00116331	48.06 SRTPpSASHEEQQE b5/y9	0.878124237	1.217524052
IPI00284016	77.33 VGGPLAVLGPSRpSSEDLAGPLPSSVPSSTTSSKPK b13/y23<br>77.33 VGGPLAVLGPSRSpSEDLAGPLPSSVPSSTTSSKPK b14/y22	0.483411729	0.411961019
IPI00885294	94.59 APpSPTDLPESEIKK b3/y12	1.095218897	0.946309865
IPI00222090	108.08 LGPSpSPAHSGALDLDGVSR b5/y15	5.270172596	4.791478634
IPI00454008	94.80 SAIpTPGGLR b4/y6	7.610964775	7.379424095
IPI00109311	138.72 EALVEPASEpSPRPALAR b10/y8	0.782189965	0.804872453
IPI00310561	108.66 VEpTPVLPPVLVPR b3/y11	3.890271187	3.738552809
IPI00123129	195.31 ETDGSEpTPEPFAAEAK b7/y10	2.279402971	1.93349576
IPI00900438	85.46 SFISSSPpSSPSR b8/y5<br>85.46 SFISSSPSpSPSR b9/y4	1.04462719	1.257072449
IPI00900438	125.18 TpSPGRADLPGSSSTFTK b2/y16	0.713234663	0.778364092
IPI00319830	46.80 RPPpSPDPNTK b4/y7	0.907872021	1.141511083
IPI00621617	133.28 VTFVDpTPGIIENR b6/y8	14.18328571	15.11098194
IPI00648313	94.80 TApSPPPPPK b3/y7	0.656178534	0.534967542
IPI00648313	55.28 VSVpSPGR b4/y4	0.742977858	0.747279286
IPI00785240	39.19 RVPSPpTPVPK b6/y5	1.10208106	1.101135373
IPI00785240	71.15 RVPpSPTPVPK b4/y7	0.738718808	0.584268093
IPI00785240	121.36 TAVAPSAVNLADPRpTPAASAVNLAGAR b15/y13	6.590248585	10.06458187
IPI00785240	55.41 TPAAAAA(ox)MNLApSPR b8/y7 b12/y3	4.672698021	2.757165194
IPI00649157	198.81 ASGQAFELILpSPR b11/y3	10.85896397	6.697172483
IPI00649157	98.01 ESVPDFPLpSPPK b9/y4	0.638173095	0.777118827
IPI00649157	85.46 RApSGQAFELILpSPR b3/y12 b12/y3	1.067178369	1.163950205

IPI00471361	36.43 LGTGGGGpSPDKSPSAQELK b8/y12	0.818988562	0.881100774
IPI00626106	166.42 NFpSDNQLQEGK b3/y9	0.806705952	0.641806185
IPI00226205	170.79 GHLLLAApTPGLAGR b8/y7	0.68735075	0.55401063
IPI00469012	37.14 ASAGPVPGAVVIAEGLHPSLPSPpTGNSTPLGTSK b24/y11	0.732137561	2.496587038
IPI00469012	50.46 AVGGAPpSPPPPVRR b7/y8	0.703671455	0.679569304
IPI00127008	46.61 IDIpSPSTFR b4/y6	0.686862171	0.794312477
IPI00649283	106.41 ASVSDLpSPR b7/y3	0.878090203	0.842149854
IPI00874522	65.28 AVPVpSPSAVEEDEDEDGHTVVATAR b5/y21	2.167450011	1.521011115
IPI00874522	138.22 AVPVpSPSAVEEDEDEDGHTVVATAR b5/y21<br>138.22 AVPVSPpSAVEEDEDEDGHTVVATAR b7/y19	2.054923415	2.129760623
IPI00896574	142.65 GPPDFpSSDEEREPTPVLGSGASVGR b6/y20<br>142.65 GPPDFSpSDEEREPTPVLGSGASVGR b7/y19	0.635078788	0.711198151
IPI00459443	73.92 SSGSLpSPGLETEDPLEAR b6/y13	1.54477489	1.255101442
IPI00459443	26.52 VSGAGLpSPSRK b7/y5	0.880207896	0.679489434
IPI00378438	112.17 AVNPT(ox)MAAPGpSPSLSHR b6/y12 b11/y7	2.11969614	1.946917772
IPI00378438	100.39 QGpSPTPALPEKR b3/y10	0.702841759	0.673131227
IPI00652758	82.58 GDLSQHApTPLPTPAVLPGDSPITPTPEQIGK b8/y24<br>82.58 GDLSQHATPLPpTPAVLPGDSPITPTPEQIGK b12/y20	3.229072332	3.116264582
IPI00652758	89.72 GDLSQHATPLPpTPAVLPGDSPITPTPEQIGK b12/y20	2.943491697	2.671375036
IPI00831423	141.67 AISEELDHALND(ox)MTpSI b13/y4 b15/y2	0.970936147	0.480093483
IPI00880644	90.47 TDGFAEAIHpSPQVAGVPR b10/y9	0.896591127	1.482336044
IPI00312128	144.27 LDLDLpTSDSQPPVFK b6/y10	6.443159103	5.571760178
IPI00877238	89.63 SEDRPpSSPQVSVAAVETK b6/y13<br>89.63 SEDRPSpSPQVSVAAVETK b7/y12	0.765558768	0.669963344
IPI00110753	144.98 AVFVDLEPpTVIDEVRpTGTYR b9/y12 b16/y5	4.930887222	9.076258659
IPI00110753	88.83 AVFVDLEPpTVIDEVRTGTpYR b9/y12 b19/y2	4.930887222	9.076258659
IPI00110753	60.78 AVFVDLEPpTVIDEVRTGTYR b9/y12	3.473395824	3.943846226
IPI00169463	62.30 I(ox)MNTFSVVPpSPK b2/y11 b10/y3	14.58024406	7.520189285
IPI00889248	164.13 QLQpSPFILDEDQAR b4/y11	5.961415768	4.067269802

IPI00123313	106.25 ATLPpSPDKLPGFK b5/y9	55.15907478	34.80770683
IPI00123313	126.13 SDpTAAAAVR b3/y7	47.94510269	33.9345932
IPI00123589	95.15 SSPPATDPGPVPpSSPSQEPPTKR b13/y11<br>95.15 SSPPATDPGPVPSpSPSQEPPTKR b14/y10	5.447118282	5.730722427
IPI00881557	49.68 IPYpTPGEIPK b4/y7	10.09185886	7.122915268
IPI00881557	103.96 VLIGGDEpTPEGQK b8/y6	20.14777565	16.13555527
IPI00404693	226.15 NLLEDDpSDEEEDFFLR b7/y10	0.709050298	0.96781987
IPI00308187	100.39 SLpTSPLDDTEVKK b3/y11<br>100.39 SLTpSPLDDTEVKK b4/y10	43.05687332	77.48855591
IPI00308187	75.38 SLTpSPLDDTEVKK b4/y10	12.88979244	18.96881676
IPI00126072	64.66 LPPLPVpTPG(ox)MEGAGVVVAVGEGVGDR b7/y20 b10/y17	3.031758547	2.699586749
IPI00227299	101.78 ISLPLPpTFSSLNLR b7/y8	35.70420074	51.64565659
IPI00227299	125.18 ISLPLPTFpSSLNLR b9/y6	2.674481869	2.110260725
IPI00751833	74.81 LLQDpSVDFSLADAINTEFK b5/y15	1.879904628	1.739447713
IPI00751833	91.73 LRpSSVPGVR b3/y7	92.36534119	57.50434875
IPI00751833	152.78 SLYSpSSPGGAYVTR b5/y10<br>152.78 SLYSSpSPGGAYVTR b6/y9	11.71202087	9.97661972
IPI00108989	84.26 QASTDAGpTAGALTPQHVR b8/y11	0.463960841	0.618706474
IPI00387422	158.19 GPLSQAPpTPAPK b8/y5	24.32490158	12.10877228

*Before each phosphopeptide is listed the PTM score, and the PTM fragment ion follows each peptide. Phosphorylated residues are indicated with a "p" and oxidated methiones with "(ox)".

Table S2 - Identified *in vitro* p38α substrates and their phosphorylation sites.

UniProt*	Recommended Name	Site(s)
Q3TXS7	26S proteasome non-ATPase regulatory subunit 1	T311
O35226-2	26S proteasome non-ATPase regulatory subunit 4	T250
Q9CQX8	28S ribosomal protein S36, mitochondrial	S60
P62281	40S ribosomal protein S11	T46
P60867	40S ribosomal protein S20	S93
P62852	40S ribosomal protein S25	T69
Q6ZWU9<br>Q6ZWY3	40S ribosomal protein S27<br>40S ribosomal protein S27-like	S27
P14206	40S ribosomal protein SA	T97
Q9CQ60	6-phosphogluconolactonase	S178
P99027	60S acidic ribosomal protein P2	S74
Q9CR57	60S ribosomal protein L14	S139
P20029	78 kDa glucose-regulated protein	T70
O54931	A-kinase anchor protein 2	S18, T19, S22
Q8QZT1	Acetyl-CoA acetyltransferase, mitochondrial	T233
Q99KI0	Aconitate hydratase, mitochondrial	S559
P68134<br>P68033<br>P62737	Actin, alpha skeletal muscle<br>Actin, alpha cardiac muscle 1<br>Actin, aortic smooth muscle	S35, T105, T108, T320
Q9QZ83	Actin-like protein Gamma	T110, T322
P17182	Alpha-enolase	S40, T41, T229, S419
P10107	Annexin A1	S37
IPI00310240†	Annexin A6 isoform b	T529
Q7TQH0	Ataxin-2-like protein	S687
P56480	ATP synthase subunit beta, mitochondrial	S128
Q9CPQ8	ATP synthase subunit g, mitochondrial	T42
Q8VCQ8	Caldesmon 1	S463, S465, T467
P35564	Calnexin	T67
P51125	Calpastatin	T624
P18760	Cofilin-1	S156
Q9D1L0<br>Q8CEW7	Coiled-coil-helix-coiled-coil-helix domain-containing protein 2, mitochondrial<br>Putative uncharacterized protein	S45
Q3UMF0	Cordon-bleu protein-like 1	T304
Q04447	Creatine kinase B-type	T35
Q8R1Q8	Cytoplasmic dynein 1 light intermediate chain 1	S421
Q9D2G2	Dihydrolipoyllysine-residue succinyltransferase component of 2-oxoglutarate dehydrogenase complex, mitochondrial	T159
Q99LC5	Electron transfer flavoprotein subunit alpha, mitochondrial	S140
Q9DCW4	Electron transfer flavoprotein subunit beta	T182, T219

P10126	Elongation factor 1-alpha 1	T269, T286, T287
P19096	Fatty acid synthase	T976, S982
Q8BTM8	Filamin-A	T1750, T2549
Q80X90	Filamin-B	S833
P05064	Fructose-bisphosphate aldolase A	T37
P13020	Gelsolin	T359, T556
Q8R5B7	General transcription factor IIF, polypeptide 1	T389, S391
P06745	Glucose-6-phosphate isomerase	S455
P17439	Glucosylceramidase	S418
P16858	Glyceraldehyde-3-phosphate dehydrogenase	T209
Q99JX3	Golgi reassembly-stacking protein 2	T417, S418
P14602	Heat shock 27 kDa protein	S180, S203, S206
P17156<br>P63017	Heat shock-related 70 kDa protein 2<br>Heat shock cognate 71 kDa protein	T178/T177
P70696<br>Q64475<br>Q6ZWY9<br>P10853<br>Q64478<br>P10854<br>Q64525	Histone H2B type 1-A<br>Histone H2B type 1-B<br>Histone H2B type 1-C/E/G<br>Histone H2B type 1-F/J/L<br>Histone H2B type 1-H<br>Histone H2B type 1-M<br>Histone H2B type 2-B	T97/T98
Q61191	Host cell factor 1	T662, S666
P06151	L-lactate dehydrogenase A chain	T309
P14733	Lamin-B1	S24
A0T1J8	LIM domain only 7	S1602
P70699	Lysosomal alpha-glucosidase	S156, T197
P08249	Malate dehydrogenase, mitochondrial	S41, S47, T309
Q9DBV4	Matrix-remodeling-associated protein 8	S423
Q9QYR6	Microtubule-associated protein 1A	T2182
P14873	Microtubule-associated protein 1B	T2300, T2301
Q62432<br>Q8BUN5<br>Q9JIW5	Mothers against decapentaplegic homolog 2<br>Mothers against decapentaplegic homolog 3<br>Mothers against decapentaplegic homolog 9	T172/T132/T136
Q9DCL9	Multifunctional protein ADE2	T27
P26645	Myristoylated alanine-rich C-kinase substrate	S140, S141, T143
P82343	N-acylglucosamine 2-epimerase	S419, S420
Q6P5H2	Nestin	T383, T389
E9Q616	Neuroblast differentiation-associated protein AHNAK	S232, T423, T551, S692, T736, T1165, S1166, S2381, S2985, T3094, S3139, S3140, T4342, T4773,

		T4775, T4779, S4890, S4905, T5169, S5194, S5195, S5325, S5566, T5567
P28656	Nucleosome assembly protein 1-like 1	T62, T64
Q8VG12	Olfactory receptor MOR245-1	T134, S137
O70400	PDZ and LIM domain protein 1	T128
Q8CI51	PDZ and LIM domain protein 5	S111
P09411	Phosphoglycerate kinase 1	S203
Q9QXS1	Plectin	T158
Q9WU78	Programmed cell death 6-interacting protein	T741
Q9Z2U1	Proteasome subunit alpha type-5	S56
Q9QYS9	Protein quaking	T243
P52480	Pyruvate kinase isozymes M1/M2	T41
Q5I1X5	RelA-associated inhibitor	S394
Q99PT1	Rho GDP-dissociation inhibitor 1	T160
Q7TQ48	Sarcalumenin	T628
Q9CZN7	Serine hydroxymethyltransferase	T420
Q8BTI8	Serine/arginine repetitive matrix protein 2	S2224, T2241
P19324	Serpin H1	S69
A2AAY5	SH3 and PX domain-containing protein 2B	S291
P38647	Stress-70 protein, mitochondrial	T87
Q62465	Synaptic vesicle membrane protein VAT-1 homolog	T122
P80314	T-complex protein 1 subunit beta	T327
O88746	Target of Myb protein 1	T196
Q62318	Transcription intermediary factor 1-beta	T498
P68369<br>P05213<br>P68373	Tubulin alpha-1A chain<br>Tubulin alpha-1B chain<br>Tubulin alpha-1C chain	T73, T80
P99024<br>P68372<br>Q9ERD7<br>Q9D6F9	Tubulin beta-2C chain<br>Tubulin beta-3 chain<br>Tubulin beta-4 chain<br>Tubulin beta-5 chain	S172
Q02053	Ubiquitin-like modifier-activating enzyme 1	T531, S835
Q922Y1	UBX domain-containing protein 1	S199, S200
O70475	UDP-glucose 6-dehydrogenase	T185, T474
Q3TW96	UDP-N-acetylhexosamine pyrophosphorylase-like protein 1	S490
Q9QY76	Vesicle-associated membrane protein-associated protein B	T158, S159
P20152	Vimentin	S55, S56, S72, T417
Q62523	Zyxin	T252

*Certain phosphopeptides matched multiple proteins and therefore all matching proteins are listed with their UniProt accession numbers and recommended names. If the site of phosphorylation varies, this is indicated by a slash (for example T12/T13).
†The IPI accession number is listed for annexin A6 isoform b as it is not found in the UniProt database.

Table S3 – Phosphopeptides identified using the whole-cell lysate *in vivo* kinase assay with p38α.

Uniprot ID	Peptide sequence with phosphorylation sites*	Abundance ratio (DMSO/SB202190)	Abundance ratio (p38β/SB202190)
P20029	169.25 IYGSGGPPPTGEEDpTSEKDEI	16621.51156	6091.5
O55022	209.45 IIKEGEEPTVYpSDDEEPKDETAR	42.93319595	3.0337
Q9EQU5	106.42 SApSPGIPK	21.22286127	1.5845
Q58A65	149.95 SASQpSpSIDKIDQEIK	13.74060486	1.0016
Q9ERU9	92.913 NRPGYVpSEEEEDDEDYEMAVK	12.36766597	3.0773
Q9JIX8	115.78 TAQVPpSPPR	10.44364609	1.0241
E9Q9F5	146.77 IYEFPEpTDDEEENK	9.525623928	0.34676
Q9R0Q7	258.41 DWEDDpSDEDMSNFDR	9.503896598	0.16796
B2RWS6	150.65 TDGKEEEEQPSTSATQSpSPAPGQSK	8.942944017	1.5173
Q8C1D8	76.759 IpSDpSESEEIPKPR	8.664760419	6.1125
P48678	146.79 NKpSNEDQSMGNWQIR	8.173273396	1.1123
F6WUK6	104.76 ASEPVKEPVQTAQpSPAPVEK	7.38989063	0.094598
Q9ERA6	199.67 TTQSIQDFPVADpSEEEAEEEFQK	7.324397568	1.5604
Q80XU3	125.31 EMIIEDVGpSEEEPEEDDEAPFQEK	6.831067696	0.94534
P48678	85.355 IpSPpSPTSQR	6.196554716	1.1772
P09405	169.13 KEDpSDEDEDEEDEDDpSDEDEDDEEEDEFEPPIVK	6.063178318	2.1139
Q9ERG0	119.53 EIpSVEEQIK	5.920312593	0.38145
P26231	260.29 TPEEIDDpSDFETEDFDVR	5.513895015	0.99434
Q80UU9	102.69 IIKPGEEPSEYpTDEEDTKDHSK	5.425347222	1.0973
P19426	152.04 SIpSEQPVVDTATATEQAK	5.243563526	0.077599
Q0VAW6	74.325 RDpSSESQIASTESDKPTTGR	5.234505863	0.87138
P97855	96.793 YQDEVFGGFVTEPQEEpSEEEVEEPEER	5.234231876	0.96794
Q149C3	47.302 VIEDGpTIEIR	5.184570718	1.0956
P16254	136.6 KSpSVEGIEPAENK	5.094503031	1.1434
P39447	82.543 pSREDISAQPVQTK	5.016806301	1.0615
Q5SF07	152.94 ISYIPDEEVSpSPpSPPHR	5.012028869	1.0268
O08709	170.79 DINAYNGEpTPTEK	4.964257347	38.856
O35344	130.29 NVPQEESIEDpSDVDADFK	4.956138177	0.97921

Q9CQU1	157.79 IVEPEVVGEpSDSEVEGDAWR	4.954173892	0.55521
E9Q9V4	83.53 MNSpSpSHRYISCWPR	4.753077618	0.076036
E9Q616	73.918 VPDVDISpSPGINVEAPDIHMK	4.560171462	12.46
P97825	233.14 SNpSSEASSGDFIDIK	4.481089801	0.94258
Q8BTM8	151.95 CGQSAAVApSPGGSIDSR	4.200092402	0.24551
P70271	145.29 SpSVSGISIEDNR	4.115903852	0.76518
Q9Z1D1	178.06 GIPIPTGDpTSPEPEIIPGDPIPPPK	4.061243553	1.2375
F8WHU5	61.862 IDpSSEMDHpSENEDYTMSSPIPGK	4.060089322	0.78106
E9Q6R7	137.05 AAQApSINAINDPIAVEQAIQEK	4.049730693	0.69808
Q8CHW4	109.24 AGpSPQIDDIR	3.879577902	1.3275
F8WJE0	74.665 pTPPpSTPPATANISADDDFQNTDIR	3.871017691	0.51234
Q8C0E3	98.407 GIGpSNEDGIQK	3.827458185	0.80299
Q3TUQ5	113.77 GFpSDSGGGPPAK	3.55707324	0.16349
E0CYH7	154.88 SNpSNSSSVITTEDNK	3.483713639	0.95428
Q6P5H2	56.488 VSQVpSIEpSIEKENVQSPR	3.480561066	0.56402
Q6P4S8	120.63 RDpSTEAPKPESpSPEPPPGQGR	3.458532199	1.4365
Q8K1Z0	81.696 YTDQpSGEEEEDYESEEQIQHR	3.417868617	0.022806
O08784	58.487 KIpSGDIEAGAPK	3.383636733	0.78798
E9QAS5	110.51 KMpSQPGpSPSPK	3.341687552	0.69177
G3XA17	87.323 FpSPTMGR	3.299567757	1.0537
Q6NXI6	213.51 DVEDMEIpSDVEDDGSK	3.227263926	1.8832
O08582	99.407 pSRSPVDSPVPASMFAPEPpSSPGAAR	3.167764825	1.1349
P17182	199.37 AAVPSGASTGIpYEAIEIR	3.158060951	1.0684
P97868	105.28 WDKDDFEpSEEEDVK	3.113518899	0.69786
F8WHT3	122.63 IKFpSDDEDEEDVVK	3.09645456	0.65508
Q61029	178.06 SSpTPIPTVSSSAENTR	3.095687707	1.2507
P70288	64.779 MIPHAPGVQMQAIPEDAVHEDpSGDEDGEDPDKR	3.093102382	0.87477
P97496	114.84 NTEKEQDpSDVSEDVKPEEK	3.03729802	0.52185
E9QN87	69.152 pSRpSpSSVGSSSSYPISSAGPR	3.023797285	0.8572
G3X8Y3	195.1 ITVNGDpSSAETEEIANEI	3.021787085	0.57215
P42208	148.06 IYHIPDAEpSDEDEDFKEQTR	2.974331519	0.8685
Q8BGD9	252.38 SQSpSDTEQPpSPTSGGGK	2.950374698	0.74824
Q1HFZ0	101.53 FQQPPQPEGEEDApSDGGR	2.919793279	1.3248

B7ZNU9	87.498 GPAGEASApSPPVRR	2.917748665	1.0515
P58871	226.74 SSGpSIpSPGIETEDPIEAR	2.893099957	1.4368
Q68FE6	112.51 HTSpSPEVVAEDR	2.841151234	3.1085
P14602	107.57 SPpSWEPFR	2.82382176	0.56086
O35126	77.221 TEQEIPRPQpSPSDIDpSIDGR	2.815949538	0.63225
Q9D6Z1	180.9 pSSPKEEVASEPEEAApSPTTPKK	2.812860398	0.96562
Q91W89	72.34 RIpSNpTDGIPR	2.772925159	0.84856
P26231	196.71 TpSVQpTEDDQIIAGQSAR	2.763652443	0.84342
Q64727	107.41 GWIRDPNApSPGDAGEQAIR	2.7451411	0.70676
P47713	299.57 HIVSNDpSSDpSDDEAQGPK	2.723608236	0.60442
E9QA15	64.298 SGGRApSGDKEAEGAPQVEAGK	2.682259535	0.87382
Q8CDT6	73.632 ENIIEPpSREDR	2.650059626	1.3821
E9QLZ9	115.56 NpSRPpSSPVNTPSSQPPAAK	2.636018558	0.37697
Q8BKT7	114.74 AIFKPPEDSQDDEpSDpSDAEEEQTTK	2.609943886	0.60725
Q8K3A9	118.31 AINAEpTPKSpSPIPAK	2.608242045	1.2371
Q64727	145.31 GQGApSPVAMQK	2.607154031	0.80933
Q8BTI8	59.975 SGpSSQEIDGKPpSASPQER	2.597267674	0.63374
Q8C2Q7	124.29 HTGPNpSPDTANDGFVR	2.539102173	3.0709
E9QN87	189.87 SpSpSVGpSSSSYPISSAGPR	2.52538007	0.84552
D3YYI8	100.29 MIPHAPGVQMQAIPEDAIPEEpSGDEDEEDPDKR	2.524360075	1.1499
E9Q616	168.5 IRpSEDGVEGDIGETQSR	2.515280328	1.2671
E9QLK0	122.79 AGEQQIpSEPEDMEMEAGDTDDPPR	2.486201581	1.4383
Q5EBP8	110.08 SEpSPKEPEQIRK	2.471882338	1.0024
Q61083	133.26 AQpSYPDNHQEFTDYDNPIFEK	2.452844073	0.86593
E9Q616	151.79 GDIGASpSPSMK	2.422011238	0.7262
Q6DFZ1	136.39 APSSpSSPGpSPMASSPSK	2.421717967	1.3408
Q8K2C9	132.79 WIDEpSDAEMEIR	2.365967917	0.67066
Q9Z0P4	114.55 SETIVNAQQpTPIGpTPK	2.346922012	0.64853
E9Q616	178.72 GGVTGpSPEApSIpSGSKGDIK	2.346316283	0.80598
Q8BTI8	108.34 RSpSSEIpSPEVVEK	2.336339423	0.95544
P43276	142.45 SETAPAETAAPAPVEKpSPAK	2.325202874	1.0141
Q3TVI8	122.08 GREPpSSSQPVVPVDVEDQAK	2.310909805	0.76217
Q6P5B0	166.28 GDSIEEIIADpSEDEDEEEER	2.305634972	0.925

Q64511	111.84	VKApSPITNDGEDEFVPpSDGIDKDEYAFSSGK	2.295684114	1.8024
E9PX78	180.63	AKPAAQSEEETATpSPAASPpTPQpSAERpSPSQEPSAPGK	2.289691807	1.1449
O35379	115.57	GSpSQIDVNEEVEAIIVK	2.283000776	0.6274
E9Q8G4	60.621	GMPPGIQGQSVSSGSSEIKpSDDEGDENIQDTK	2.260551122	0.079554
Q9DCD5	77.746	KDpSITQAQEQGTVIS	2.256012273	0.92359
Q52KI8	81.625	RIpSPpSApSPPR	2.249162187	1.023
E9Q983	56.819	ARYPpSGSEIPVVEDEEKVDER	2.249162187	0.71241
Q8C2Q3	60.91	TRIpSPPR	2.220445866	1.1252
A2BDX0	78.088	IM(ox)HDApSDpSEVDQDDVVEWK	2.219608017	2.14
Q61029	121.82	GPPDFpSpSDEEREPpTPVIGSGASVGR	2.208334253	0.67348
Q8BMQ2	94.45	IIIVDpSPGMGDGEDEQQEEGTSK	2.197753896	1.158
Q91YM2	56.527	NEEENIYpSVPHDSTQGK	2.164642726	1.1029
F8WIP8	112.44	ISHEIESSpSSEVN	2.157916316	0.98433
E9PYF4	119.54	SRpSTTEINDPIIEK	2.15132414	0.70413
Q5SNZ0	198.16	SSpSQENIIDEVMK	2.150029025	1.1292
O54724	202.25	ATEEPpSGpTGSDEIIK	2.142336861	0.75172
Q80TT4	66.636	ApSPAIGSGHHDGSGDSIEMSSIDR	2.12630236	0.91587
Q99J36	115.87	FIDKDQQPpSGpSEGEDDDAEAAIKK	2.119227753	0.86709
P18608	129.82	QADVADQQTTEIPAENGETENQpSPApSEEEKEAKSD	2.086767805	1.0805
Q5EBH1	50.752	CANCKFTCHSECRpSIIQIDCR	2.075550021	0.87325
Q9CWY8	105.38	EAEDVIWEDpSEAEEDPERPGK	2.075550021	0.87325
P39447	94.542	VQIPVSHPDPEPVpSDNEDDSYDEEVHDPR	2.051113755	0.30289
Q8R1A4	149	SRpSISNpSNPDISGTPpTSPDDEVR	2.022858299	0.61098
Q99J27	121.38	RDpSVGGEGDREVIIGDAGPGDIPK	2.02052857	1.0595
P58871	214.66	SIpSSGFSPEEAQQQDEEFEKK	2.020161209	0.94867
Q05D44	178.32	TARPNSEAPIpSGpSEDADDSNK	2.011748612	1.0565
Q8C1D8	178.32	KAAVIpSDpSEDDAGNASAK	1.997882245	0.02664
Q61687	96.666	ITIpSDGEpSGEEKPTKPK	1.995609659	0.71343
Q8VDJ3	209.66	VApTINSEEENDPPTYK	1.994256541	0.81537
P16460	99.566	APNpSPDVIEIEFK	1.990921398	18.818
Q8BH64	162.43	GPDEAIEDGEEGpSEDDAEWVVTK	1.98298598	1.1434
Q02248	93.495	TpSMGGTQQQFVEGVR	1.971375626	0.80444
P62960	82.885	NYQQNYQNpSESGEKNEGSESAPEGQAQQR	1.945941739	1.2884

Q3TDQ1	102.33	ENPPVEDpSpSDEDDKRNPGNIYDK	1.945828144	0.98807
Q9ESX5	105.46	RDpSEpSESDETPTVPQIK	1.940730103	0.71792
Q78PG9	98.943	VENMSSNQDGNDpSDEFM	1.940579457	0.75608
Q3TYK4	157.68	TDpSREDEIpSPPPPNPVVK	1.92008602	0.7563
Q9CSN1	104.21	GPPpSPPAPVMHpSPSR	1.918833349	0.50816
Q05CL8	199.29	pTASEGpSEAETPEAPKQPAK	1.916039164	0.94207
Q80X50	215.06	STSAPQMSPGpSSDNQSSpSPQPAQQK	1.914535151	0.67551
Q6P5H2	89.049	QESIKpSPEEEDQQAFR	1.902768528	0.92668
Q9JIX8	106.26	SIpSPISGTTDTK	1.900418092	1.0101
Q61686	95.094	SpSFSNSADDIK	1.896777375	0.62902
Q7TSC1	73.11	IKFpSDEEDGRDpSDEEGAEGHK	1.874027848	0.83324
Q9JI10	132.76	EIEEEEENpSDEDEIDSHTM(ox)VK	1.872799461	1.1061
Q569Z6	78.272	pSPVGKpSPPATGSAYGSSQK	1.853361999	0.49006
P62996	76.326	RpSPpSPYYSR	1.850104531	0.78908
Q9JHF5	199.41	IIASPDASTIENpSWSPDEEK	1.845086535	0.57215
O08539	224.67	SPpSPPPDGpSPAATPEIR	1.840468215	0.81505
F6R9D8	54.514	RGPNYTSGYGTNSEIpSNPpSETESER	1.840332732	1.0421
Q7TSC1	105.5	TApSETRpSEGSEYEEIPK	1.839317245	0.71032
Q9EQC8	129.77	IAAPEIQKGDpSDpSEEDEPAK	1.833920188	0.99815
Q62018	188.86	KGGEFDEFVNDDpTDDDIPVSK	1.831803777	1.168
Q6PGF5	62.1	GKPGPDTQSEDIEEEEVKEEpTDPpSEEESAR	1.828788793	1.1905
P19426	167.32	SMpSADEDIQEPSR	1.819803097	0.99136
Q14AX6	116.96	NNpSPAPPQPAPVK	1.815442151	0.83844
F8VPY3	212.95	EGpSPAPIEPEPGASQPK	1.815244423	0.8924
Q5I012	160.43	ISVQDPVVVVAEDpSQEK	1.799337844	0.79529
P70255	100.45	TEMDKpSPFNSPpSPQDSPR	1.797591228	0.0702
Q8C8U0	186.97	SQpSTTFNPDDMSEPEFK	1.794848784	0.82177
Q8CI75	110.55	RPGIEKApSDEEPED	1.794204719	0.9879
Q99LL5	95.435	IQEEGGpSEEEEAGNPSEDGMQSGPTQAPPR	1.775725828	1.0044
Q9CWU4	178.51	IIPEGEETVEpSDDDKDER	1.770694998	0.81544
Q62093	124.08	pSKpSPPKSPEEEGAVSS	1.760873393	0.72086
F6YZ95	59.975	SASAPTIAEpTEKETAEHINIAGTSR	1.76056338	0.83512
Q922D4	187.39	IQQFDDGGpSDEEDIWEEK	1.758489106	0.69979

P47856	125.61	VDpSTTCIFPVEEK	1.756573978	0.62448
Q8R550	115.91	pSIEVENDFIPVEK	1.749444551	0.83033
Q91XV3	75.887	AEPEKpSEGAAEEQPEPAPAPEQEAAAPGPAAGGEAPK	1.742828262	1.1817
P97310	183.74	GIIYDpSpSEEDEERPAR	1.74206923	0.96586
Q9Z2D6	261.28	AETSESSGSAPAVPEApSASPK	1.736623656	1.0058
P20152	89.296	SpSVPGVR	1.736171395	0.75239
E9Q616	213.03	SNpSFSDEREFSAPpSTPTGTIEFAGGDAK	1.734876217	3.1973
C7G3P2	137.86	EQAEMDDADNpSEKpSVNEENGEVSEDQSQNK	1.733883552	0.98245
Q5U3K5	71.592	NISIpSpSEEEAEGIAGHPR	1.72887744	0.91308
Q52KI8	107.97	QpSPpSPSTRPIR	1.725506436	0.81347
Q52KI8	100.41	KVEIpSEpSEEDKGSK	1.723543606	0.87793
Q8R1Q8	212.79	KPASVSPTpTPTpSPTEGEAS	1.721081528	0.74512
Q9JIK5	254.53	EIITEEPpSEEEADMPKPK	1.717770334	0.92401
Q9JIK5	97.271	SNSSDAPGEEpSSSETEKEIPVEQK	1.698946653	1.0758
Q8K3W3	135.09	GTVTGERQpSGDGQESTEPVENK	1.697994668	1.0195
Q61699	73.389	NIQQDNSEAGTQPQVQTDGQQTSQSPPpSPEITSEESK	1.696899764	0.40343
Q9ET54	142.1	IApSDEEIQGTK	1.693594824	0.68673
E9PWZ7	106.63	SAEEVPDDVDMEGNKEpSDDpSDEEYDITEK	1.689331869	0.88282
Q05D44	202.35	TSFDENDpSEEIEDKDSK	1.674060434	0.91823
B2RQC6	100.55	IHRApSDPGIPAEEPK	1.661709234	0.65317
A6H619	110.98	EVpSPAPATQGESR	1.658759911	0.69804
Q5DTM8	218.12	AIVVPEPEPDSDpSNQER	1.658237294	0.88664
Q3UHP6	135.81	SPpSFASEWDEIEK	1.65598556	1.4936
F8VQL9	75.682	IEPVpSPPpSPPHADPEIEIAPSR	1.652537471	1.0906
O54825	146.06	IGPGIPQDGpSDEEDEEWPTIEK	1.646036344	0.96945
Q3U3M5	68.676	KPpSPQPpSPPR	1.645955065	0.72187
Q52KI8	67.385	RRpTPpSPPPR	1.644277094	0.95199
E9PYU6	122.29	IEGDSDDIIEDpSDSEEHSR	1.641039106	0.9907
Q6P5H2	155.2	ESQEFIRpSPEAEEEEEQVM(ox)VR	1.641012176	0.92351
F8VPU2	259.18	SPDEATAADQEpSEDDISASR	1.639371137	0.99354
F8WHU5	202.96	AEpSPETSAVESTQpSTPQK	1.638968106	0.94864
Q05D44	237.59	SVPTVDpSGNEDDDSSFK	1.627524698	0.95236
B2RY56	87.447	IGASNpSPGQPNSVK	1.623956608	0.87276

E9Q8Z8	75.739 GpSIApSIDSIRK	1.622507423	0.91181
Q9JKB3	86.756 pSRPINAVSQDGK	1.619118552	0.85579
P39447	134.61 pSVApSSQPAKPTK	1.613527817	0.84849
P51859	124.17 NpSTPSEPDpSGQGPPAEEEEGEEEAAKEEAEAQGVR	1.612227131	1.0896
Q7TPW1	153.34 TVpSQESITPGK	1.609709769	0.85188
Q80U72	73.126 AHEEEEEEEEENRDEEEGEApTpTEEDDKEEAVASAPSVK	1.609372988	0.14591
Q9CQF4	66.372 EADEEDpSDEETSYPERpSEQEEEIESEPGVAK	1.60774289	7.5367
Q3UPL0	194.08 DSDQVAQpSDGEEpSPAAEEQIIGER	1.606322485	0.8903
Q52KI8	71.295 AKpSPpTPpSISPARNpSDQEGGGK	1.604132245	0.62202
Q8CCP0	84.327 NPYIIpSEEEDGDGDASIENSDAEAPK	1.591393743	0.91654
P52479	141.83 SDIIEDEEIEDTGKGpSEDEWEQVGPK	1.587276392	0.90199
Q91YL3	113.86 YFGTDAVPDGpSDDDEAATVG	1.585162875	0.97715
Q3UJB0	178.53 SSIGQSApSETEEDTVSISK	1.581352689	0.49272
E9Q616	120.57 IQGpSGVpSIASK	1.579329732	0.63374
Q3TUQ5	157.2 SIpSPGKENINSQEVEK	1.578531965	0.54821
Q8BTM8	174.52 IPEISIQDMTAQVTpSPSGK	1.577336825	0.57564
Q7TPW1	115.39 KREDEEEEEGpSIVNGSTTEDEEQTR	1.565214669	1.2048
Q80XU3	150.61 ATVpTPpSPVK	1.553615263	0.83681
Q8BX17	118.51 APpSQPPpSPTEER	1.541568392	0.81169
Q64213	91.276 TGDIGIPPNPEDRpSPSPEPIYNpSEGKR	1.537279016	0.83791
A2AI52	184.22 SYpSSPDITQAIQEEEKR	1.536381514	0.63327
E9QPD4	184.78 TGpSNIpSGASSDVSIDEQYK	1.53230873	0.81939
P31750	120.41 pSGSPSDNpSGAEEMEVSIAKPK	1.52434377	0.87021
E9QN52	152.71 RGpSGDTSSIIDPDTSISEIR	1.52121332	0.53339
A2AJI0	148.35 AAEEKEPAAPApSPAPpSPVPpSPTPAQPQK	1.519710647	1.0678
Q8BTI8	233.63 NSGPVSEVNTGFpSPEVK	1.518948887	0.97391
E9Q4S2	147.09 YGIQDpSDEEEEEHPPK	1.51388994	0.7104
Q91UZ1	68.42 ANVpTPQSpSSEIRPTTTAAIGSGQEAK	1.499003163	1.0382
Q9DBY8	181.58 ESIPIDIpSDDQSNSK	1.498217122	0.83142
Q8K3X4	109.35 KApSPEPPDSAESAIK	1.482030382	0.49001
P39447	84.942 AVPVpSPSAVEEDEDEDGHTVVATAR	1.481064589	0.55536
P39053	77.776 RpSPTSpSPTPQR	1.479618258	0.75249
P51859	209.71 KGpSAEGpSpSDEEGKIVIDEPAK	1.478590017	0.7763

Q9JMH9	123.4 NKIEGDpSDVDpSEIEDR	1.47579693	1.1793
Q3TPJ8	76.21 EAEAIIQSMGITTDSPIVPPPMpSPSSK	1.47275405	1.9899
Q99L43	63.138 IDGETApSDSESRAETAPIPTSVDDTPEVINR	1.461326007	1.658
Q3TKT4	73.865 KAENAEGQTPAIGPDGEPIDEpTSQM(ox)pSDIPVK	1.458555155	0.50021
Q9ET78	69.639 RpSDSAPPpSPVSATVPEEEPPAPR	1.455752406	0.87896
Q9D0L8	193.87 ASVASDPEpSPPGGNEPAAASGQR	1.454820548	0.74633
F6ZFU0	105.2 ATAPQTQHVpSPMR	1.449779633	0.95593
Q6ZQ58	88.976 VEPAWHDQDETSpSVKpSDGAGGAR	1.449212353	0.89189
Q9JKX4	79.382 HIVNGAKPNTEpSEEIpSpSEDDEIVGEK	1.447827535	1.3948
O55106	250.79 FIESAAADVpSDEDEDEDTDGR	1.447659858	0.99518
P42128	126.63 EGpSPIPHDPDIGSK	1.447366517	0.7794
P70268	161.51 TDVSNFDEEFTGEAPTIpSPPR	1.44221062	0.84246
E9PUU4	119.31 APpSQPPpSPTREER	1.436554568	0.76215
E9Q8T1	70.986 IDNTPApSPPRpSPTEPSDTPIAK	1.435750179	0.89002
Q5SUF2	70.555 ESDTKNEVNGTSEDIKpSEGDTQpSN	1.432131298	1.6292
Q8R409	133.75 GQNGEDISTGGApSPSAEGEPMSESIVQPGHDSEATK	1.428693888	1.0305
Q9QYC0	80.786 SPPDQSAVPNpTPPpSTPVKIEEDIPQEPTSR	1.424927685	1.5264
Q8CGC6	53.522 VEEQVEDpSDDEEDDDpSHDDEEERESTIASPVSVHK	1.414607234	1.4469
P14873	195.77 SIMpSpSPEDITK	1.413048086	0.81855
Q6P5H2	106.82 APIVGpSPVHIGPSQPIK	1.409741312	0.83615
F8WGL8	120.33 EGINPGYDDYADpSDEDQHDAYIER	1.409105641	1.0248
Q52KI8	103.44 APQTSpSPPPVR	1.399364688	0.88158
Q8BHK9	167.71 IIpSDDEDEDEEDAFK	1.398444929	0.77821
E9PYT0	98.165 GGIDNPAIpTSDQEVDDKK	1.396999246	0.9343
Q2KN98	125.22 SpSTSSEPTPTVK	1.394097392	0.84283
P14873	146.78 SPSIpSPSPPpSPIEK	1.391730338	1.0726
P97801	228.08 GTGQpSDDSDIWDDTAIIK	1.391691601	0.58099
Q61029	74.579 QNGSNDpSDRpYSDNDEDSKIEIK	1.389622301	0.85628
E9QN92	142.45 RIVDpSDGpSIAEVPK	1.387578398	0.93677
Q8C1Z8	73.022 IGTpSDGEEERQEPR	1.387270407	1.3856
Q8BTI8	129.05 SEQPISQVIPpSISPEHK	1.384888101	0.82194
Q6ZQ58	88.329 ESPRPPAAAEAPAGpSDGEDGGRR	1.382972838	0.7906
P58871	126.16 VPpSpSDEEVVEEPQSR	1.377979881	0.86231

D3Z428	50.354 EIPDGpSVAAIK	1.374891727	0.78049
Q6ZPZ3	164.15 TGTGpSPFAGNpSPAR	1.374400418	1.0497
Q9CXL3	98.703 TSEAGPDKETASTIVQEApSPEISPEER	1.374268202	0.55319
E9PW34	58.98 NTTAIFpSQFVSGNNR	1.371083842	1.0335
F6ZFU0	129.08 GApTPAEDDEDKDIDIFGpSDEEEEDKEAAR	1.366456232	0.88664
Q8BTI8	143.42 VSpSPVIETVQQR	1.356410396	1.0265
Q6P9Q4	61.435 KPVpSPPpSPK	1.356410396	0.75471
Q6ZQ58	101.64 AVpTPVPTK	1.35525228	0.70189
Q9D7S7	157.58 YFQISQDEDGpSESED	1.35367455	1.1246
Q3UHJ0	58.32 VGSIpTPPSpSPK	1.351917695	0.69101
P26645	194.55 AEDGAAPpSPSSEpTPKK	1.348654043	0.92365
Q9CZ44	119.74 HpSGQDVHVVIK	1.344827122	0.88637
Q8BKT3	116.45 TAEIpSpTDEEEGTHTITGSK	1.342894744	0.74662
Q3U6P5	132.42 MESEAGADDpSAEEGDIIDDDDNEDRGDDQIEIK	1.337399026	1.2743
F7BGY9	239.75 RETVVESQSSQpSPpSPK	1.333671197	0.92668
Q9JLM8	109.65 SGKpSPpSPSPpTSPGSIR	1.328462305	2.1722
Q7TQ95	122.01 ADpSVPNIEPSEESIVTK	1.327545236	1.0023
E9QPP9	133.86 AQpSTDpSIGTSSSIQSK	1.326154417	0.65223
E9QK89	199.41 VIIAADpSEEEGDFPSGR	1.324415602	0.91554
Q3TWW8	89.805 SHpSPIPAPPSK	1.323907115	0.90688
P46935	65.472 RQIpSEDVDGPDNR	1.318061395	1.2014
Q3TYK4	89.028 TDpSREDEIpSPPPPNPVVK	1.316777056	0.81995
D3YVW2	61.583 AAVEDINPADDPNNQGEDEFEEAEQVREENIPEEpSEEQK	1.315512524	1.2438
O08784	112.23 SAEPIANTVIApSETEEEGNAQAIGPTAK	1.315062728	1.5111
Q9ERA6	86.539 GAAEEADpSEDpSDAEEKPVKQEDFPK	1.312870065	0.74778
Q68FM7	88.507 pSIENPpTPPFTPK	1.307104111	0.71157
P20152	120.77 DGQVINEpTSQHHDDIE	1.303627997	1.1029
B2RSI6	38.293 QpYpSFEAIR	1.303458074	0.84368
E9PX78	156.47 GHPSAGAEEEGGpSDGpSAAEAEPR	1.302693971	0.94401
F7AA26	101.39 EGSEQQEPEATVEEAGSQTPGSEKPQGMFAPPQVpSSPVQEK	1.302371619	0.84395
Q6P542	218.25 QISVPApSDEEDEVPAPIPR	1.297336568	0.9398
Q9Z0P4	183.31 EPAPINGpSAAEIPATK	1.29713463	0.87279
Q3TZR9	184 TDSVIIADQpTPpTPTR	1.292841536	0.46755

Q9D6Z1	278.4 EEIApSDIEEMATSSAK	1.290772269	0.80726
A2ARP8	75.524 TEATQGIDYVPSAGTIpSPpTSSIEEDKGFK	1.29033923	1.4228
E9Q942	101.86 EIVGDTGSQEGDNEQPpSGSEpTEEDPSASPQK	1.28093457	0.87594
Q80WJ7	66.777 SQEPISNDQKVpSDDDKEK	1.280622895	0.85604
Q8K4Z3	61.167 RGpSETMAGAAVK	1.274193754	0.78689
E9PW44	155.42 GIpSPIVFDR	1.268456035	0.90629
Q9WVA4	90.861 NFpSDNQIQEGK	1.262291564	0.53262
E9Q983	73.834 KIpSVDNNTSATDYK	1.262148176	0.92537
Q64337	165.19 pSRITPTpTPESSSTGTEDK	1.246634088	0.80949
P62960	175.39 AADPPAENSpSAPEAEQGGAE	1.246494235	1.0085
Q3TIR3	148.85 GIMAGGRPEGQYpSEDEDpTDTEEYR	1.242128014	0.97609
P28667	144.29 GDVTAEEAAGApSPAK	1.241865779	0.9805
O54774	211.78 HSpSIPTEpSDEDIAPAQR	1.23845144	1.061
O70251	176.46 YGPSSVEDTTGSGAADAKDDDDIDIFGpSDDEEESEEAKK	1.238052791	1.0216
Q8BGT6	77.124 KPpSPpSASPTVR	1.232559286	0.92506
Q8CJ53	123.4 VPpSDSpSIGTPDGRPEIR	1.23170912	1.0834
Q60876	76.326 NpSPVAKpTPPK	1.230345235	0.78058
Q1HFZ0	217.86 EGVIITNENAApSPEQPGDEDAK	1.22600103	0.9002
Q9Z1Z0	137.64 IKDIGHPVEEEDEpSGDQEDDDDEIDDGDKDQDI	1.225910852	1.2867
E9Q8D0	224.64 EFGDGpSDENEVEDQEPR	1.22577561	0.79898
E9Q0N2	40.837 pSENGpSICGKGVpTVSDIEPAQSYTApTVpTpTVFKDIK	1.224934772	0.14503
Q9JIX8	218.12 SQpSPpSPPPIPEDIEK	1.222434111	0.84552
P54103	146.77 NApSTpSFQEIEDKK	1.22083725	0.6481
Q99M51	117.35 RKPpSVPDTASPADDSFVDPGER	1.214152158	0.99456
Q9CZ44	76.262 KpSPNEIVDDIFK	1.210595129	0.98361
Q8R1B4	80.746 QPIIIpSEDEEDTKR	1.210082407	1.017
Q9WVJ0	9.2633 pSPDGGNpSSGQSENCpSpSpSpSR	1.206418145	0.74675
A2AU91	100.29 EIIEEGPQVQPpSSEPEVSSTQEDIFDQSSK	1.206258067	1.2835
P14873	164.99 QGVDDIEKFEDEGAGFEESpSETGDYEEK	1.192122455	0.90397
A2A4P5	121.32 RPPPAMDDIDDDpSDS	1.188128223	0.77223
D3Z4V2	87.138 DHSPTPpSVFNpSDEER	1.186591516	0.89739
P14602	136.5 QIpSSGVSEIR	1.181991182	0.89535
Q8BTI8	84.605 SSpSPVTEITAR	1.177010628	1.0746

P51612	137.45 SEAAAPHAAGGGIpSpSDEEEGTSSQAEAAR	1.175516346	0.82674
P46935	140.48 RPSPDDDIpTDEDNDDMQIQAQR	1.17362627	1.0099
P14873	153.19 VIpSPIRpSPPIIGSESPYEDFISADSK	1.173116854	1.4892
A2AU91	133.41 TEEDRENTQIDDTEPIpSPVSNSK	1.172937975	1.1177
Q8C9B9	85.387 NTTKPETIPDMEDpSPPVpSDSEEQQESVR	1.172827923	1.2704
P36916	63.682 EEQpTDpTSDGESVTHHIR	1.17241541	1.0595
B1AQD9	142.2 SAEPTREPGAEAEpSGpSESEPEPGPGPR	1.168715815	0.61293
P47811	240.85 HTDDEMTGpYVATR	1.164076596	7.1597
P35601	176.83 IIYDpSDpSESEETVQVK	1.163372384	0.86052
P70670	112.35 VQGEAVSNIQENTQTPTVQEESEEEEVDEpTGVEVK	1.162196086	1.165
Q9R0P4	232.02 pSASPDDDIGSSNWEAADIGNEER	1.154827757	0.99153
E9Q616	164.48 AEpSPEMEVNIPK	1.149319603	0.70898
G3X8U4	115.5 IQEEVPpSEEQMPQEK	1.148659514	0.90816
F8VQC1	63.979 AVpSSPPTpSPRPGSAATISSSASNIVPPR	1.148105626	0.68239
Q62261	242.1 GDQVSQNGIPAEQGpSPR	1.143889912	0.84994
Q14AR7	58.98 VITANpSNPSpSPSAAK	1.140680073	0.03546
Q99JF8	101.85 ETNVSKEDTDQEEKApSNEDVTK	1.139042976	1.0294
A2A484	111.43 SNpSPVSEKPDPTPAK	1.133504115	0.86477
Q3UH70	164.2 TKPPPTYEpSEEEDK	1.12847712	0.78809
E9QQ56	119.08 KEpTPpSPEMETAAQK	1.126620926	0.77165
Q9CZX7	90.907 SPIIpSASHSGNVpTPTAPPYIQESSPR	1.123103359	1.603
Q810A7	95.519 YMAENPTAGVVQEEEEDNIEYDpSDGNPIAPSKK	1.121025963	0.93787
Q80XU3	78.088 TPSPKEEDEEAEpSPPEKK	1.120510953	0.84756
O35218	120.33 EADIDpSpSDEpSDVEEDVDQPSAHK	1.119557551	1.0006
E9Q9C3	119.39 TSpSVVTIEVAK	1.119181654	0.66233
Q6PDG5	170.5 DMDEPpSPVPNVEEVTIPK	1.117355889	0.98663
E9QPU9	113.65 ITVEKDPDSAIGIpSDGEpTSPSSK	1.116906615	1.0768
Q6NZR5	127.58 IIEPIDIpSGGDEDEGEAAGGPR	1.112099644	0.71401
E9Q7G0	226.57 TQPDGTSVPGEPApSPISQR	1.112000712	0.71022
Q569Z6	176.81 WAHDKFpSGEEGEIEDDEpSGTENREEK	1.102997948	1.0258
Q61687	130.4 YVEpSDDEKPTDENVNEK	1.098551011	0.88256
Q60875	57.806 IQDSpSDPDTGSEEEVSSRIpSPPHSPR	1.096623496	0.57667
Q8R3N6	84.499 TGEDEDEEDNDAIIKENEpSPDVR	1.090940827	0.75509

E9Q1W3	46.819 SIYpSSNIpYK	1.090132124	0.94259
Q922Y1	67.478 SpSPPATDPGPVPpSSPSQEPPTKR	1.085340308	0.79333
Q62314	143.57 VSGPSpSSENQEGTITDSMK	1.0801469	0.7757
Q07113	88 AEAISSIHGDDQDpSEDEVITVPEVK	1.080065236	0.86009
Q65Z40	61.276 VEEEDTGDPFGFDpSDDESIPVSSK	1.080065236	0.63882
A2ARP8	139.11 AEIEEMEEVHPpSDEEEEETK	1.078027641	1.0027
E9Q0N0	142.45 IPEEPSpSEDEQQPEK	1.077969537	0.86083
Q8K0L9	85.596 DGQAEPAQPEQAAEAPAESSAQPNQIEPGApSSPER	1.077249566	1.106
P58871	160.64 MQAEpSQSPTNVDIEDKER	1.074183084	0.82076
Q5U4C3	121.26 REVIYDpSEGIpSADER	1.070320026	0.95534
Q6PDM2	85.837 VKVDGPRpSPpSYGR	1.068764295	1.249
E9Q3T0	167.14 KEEpSEEpSEDDMGFGIFD	1.065359825	0.98113
Q8BR65	84.689 RPApSPpSSPEHIPATPAESPAQR	1.064815308	0.71873
P14873	104.38 SVpSPGVTQAVVEEHCApSPEEK	1.063626114	0.85119
Q62422	211.78 TISNAEDYIDDEDpSD	1.063162483	0.96883
Q9ERG0	139.22 SDNEETIGRPAQPPNAGEpSPHpSPGVEDAPIAK	1.057328343	1.1996
P14873	272.48 ASISPM(ox)DEPVPDSEpSPVEK	1.050309841	0.94826
Q9JMH9	143.88 FSHSYIpSDpSDTEAK	1.045773506	0.86608
E9QPD4	181.87 MIAEpSDDpSGDEESVSQTDK	1.045139578	0.86489
P14873	66.017 AEEDMDDVIEKGEAEQpSEEEGEEEDKAEDAR	1.043558116	1.2591
O08553	171.76 TVTPASpSAKpTSPAK	1.039079791	0.84136
Q5U4C3	101.32 APpSPAPAVpSPK	1.034618329	0.72651
E9Q616	223.11 IPpSGpSGPASPpTTGSAVDIR	1.034201028	0.60221
Q8K019	105.19 KAEGEPQEEpSPIK	1.031651054	0.88102
P13595	165.64 GVTASSSpSPASAPK	1.03117234	0.94347
Q8VDD5	122.63 KGTGDCpSDEEVDGKADGADAK	1.031119177	0.63616
O54774	160.77 VDIITEEMPENAIPpSDEDDKDPNDPYR	1.029590429	0.81513
E9QAS5	64.583 TPTPSpTPGDTQPNpTPAPVPPAEDGIK	1.027221366	1.4166
E9Q616	67.113 ADIDVpSGPK	1.025294003	0.83057
E9PYD5	187.16 KKEPAISSQNpSPEAR	1.01705603	0.95863
P48678	166.3 SGAQASpSTPISPTR	1.016353122	2.5815
Q80YR5	89.663 pSEPVKEEGSEIEQPFAQATSSVGPDR	1.015537727	1.0549
Q80Y81	89.266 TpSPNRIpSPK	1.010386776	0.9145

A2AMM0	141.31 GGYpSPQEGGDPPpTPEPIK	1.004732289	0.8041
Q64337	133.46 EVDPSTGEIQSIQM(ox)PESEGPpSSIDPSQEGPTGIK	1.001061125	0.98121
P58871	208.46 GEGVSQVGPGpTPPAPEpSPR	0.992358837	0.92514
E9Q616	155.43 SKGHYEVTGpSDDEAGK	0.990491284	0.81515
Q91XV3	125.45 AEGAGTEEEGpTPKESEPQAAADATEVK	0.986193294	0.81443
Q80YR5	97.095 APTAAIpSPEPQDSKEDVKK	0.984058256	0.77192
E9QMN5	95.138 RPPpSPDVIVIpSDSEQPSpSPR	0.967960507	0.79802
Q6NZR5	147.62 ASpSIEDIVIK	0.958405214	1.0873
A2ARP8	78.69 VPSAPGQEpSPVPDTK	0.954107432	0.76524
Q8BGD9	178.06 YAAIpSVDGEDEDEGDDCTE	0.948226816	0.89449
P53569	88.241 SQIDDHPEpSDEENFVDVGDDpSDDEKFTDADK	0.946969697	0.0055448
Q0GNC1	81.406 SSHQDATDPEAIWGVHQTEADpSpTSEGPEDEAQR	0.942862531	1.0872
Q80UG5	138.05 pSFEVEEIEPPNSTPPR	0.942329438	1.2455
Q52KI8	85.536 EKpSPEIPEPSVR	0.934492104	1.6208
E9PWE8	72.34 GpSPTRPNPPVR	0.93057882	0.85421
Q80X90	186.72 IVpSPGSANETSSIIVESVTR	0.92936803	0.67427
F8WHU5	221.79 METVSNASSSpSNPSpSPGR	0.929281665	0.73808
Q3TYD6	26.659 PSIQpTpSKpYFpSPPPPARSAEQpSWPHVSPCpSR	0.921998894	0.96079
Q60875	85.533 IpSPPHpSPR	0.918864284	1.067
O55091	101.5 TEEVEVEpSEEDPIIEHPPENPVK	0.915248032	0.58751
Q60876	76.326 pTPPKDIPAIPGVpTSPTSDEPPMQASQSQIPSSPEDKR	0.907029478	1.5018
P42567	150.76 INDPFQPFPGNDpSPK	0.898795614	0.71918
P26645	151.96 GEATAERPGEAAVASpSPSK	0.889205051	0.97082
Q3U2G2	123.19 MQVDQEEPHTEEQQQPQpTPAENK	0.889046942	2.7206
F8VPM4	58.885 pSGDETPGSEAPGDK	0.886524823	1.1003
Q68FF6	66.031 SQSEIDDQHDYDpSVApSDEDTDQEPIPSAGATR	0.884955752	1.1312
P27546	77.222 GQSTVPPCpTASPEPVK	0.882612533	0.85463
Q9ERG0	88.338 TSpSIPESpSPSK	0.870852565	0.98042
Q9ESX5	191.35 TVIEpSGGETGDGDNDTTKK	0.858442785	0.8757
Q8BRN9	73.405 IASEDAAIVDDDEEpSDTPAQAPIAK	0.838574423	0.5404
P35564	174.9 AEEDEIINRpSPR	0.829393713	1.0018
A2AJT5	81.967 SKFDpSDEEDEDAENIEAVSSGK	0.824538259	0.56058
Q6P5H2	176.33 IVEKEpSQEpSIKpSPEEEDQR	0.823858955	0.96047

Q8VDM4	82.477	DKpTPVQSQQPSATTPpSGADEK	0.821220333	0.84904
Q569Z6	56.817	ERpSPAIKpSPIQSVVVR	0.816126663	0.91786
G3X9B8	100.97	AKEVENEQTPVpSEPEEEK	0.811490708	0.31779
Q9D6Z1	223.65	pSSPKEEVASEPEEAApSPTTPKK	0.803987779	0.90468
Q9Z1W9	194.77	TEDGDWEWpSDDEMDEK	0.801603206	0.82104
Q9CX60	124.08	IPpSIVVEPTEGEVESGEIR	0.786101722	0.96326
E9PVK0	75.773	GEEDSDVSIAPAVQQMSpSPQPADER	0.784498313	0.81952
P25206	99.021	DGESYDPYDFSEAETQMPQVHpTPK	0.772857253	0.85421
F2Z408	102.73	TApSAGTVSDAEAR	0.756143667	0.58704
Q6NVE8	94.259	EYVSNDApTQSDDEEKIQSQQTDTDGGR	0.754204691	1.1809
Q9D0F4	115.5	IGEIGAPEVWGIpSPK	0.75289866	0.71343
P11499	215.48	IEDVGpSDEEDDSGKDKK	0.751484181	0.98307
Q3U6P5	137.8	QADISFpSSPVEMK	0.748783227	0.79534
E9QQ56	216.26	AQDKPEpSPSGSTQIQR	0.746380057	0.85925
Q52KI8	125.97	YpSPpSPPPK	0.741729714	0.72454
Q9QWY8	66.022	TIpSDPPpSPIPHGPPNK	0.72285673	0.83249
G3UW69	162.32	KEEpSEESDDDMGFGIFD	0.717257208	0.69268
Q99JF8	200.67	NIAKPGVTSTpSDpSEDEDDQEGEKK	0.715307582	0.84967
E9Q616	214.54	pSSKApSIGSIEGEVEAEASSPK	0.711085828	0.84177
Q9Z1R2	168.09	ENApSPAPGTTAEEAMSR	0.699741096	0.9284
O08539	133.28	VNHEPEPASGApSPGATIPK	0.699056274	0.90885
P35564	218.72	QKpSDAEEDGVTGSQDEEDpSKPK	0.697885407	0.76553
P14873	128.95	EEQpSPVKAEVAEK	0.692664681	0.9247
Q8K019	160.81	FHDpSEGDDTEETEDYR	0.688752669	1.0128
Q62261	79.659	RPPpSPDPNTK	0.687143544	0.85045
Q8BTM8	86.856	FNEEHIPDSPFVVPVApSPSGDAR	0.674490759	1.6864
P14873	250.26	ESpSPIYpSPGFSDSTSAAK	0.670600858	0.80565
Q8BTI8	127.71	SAVRPpSPpSPER	0.668270516	0.97679
E9PX78	115.89	pSPSQEPSAPGK	0.666666667	0.66455
Q9Z1Q5	108.64	VIDNYITpSPIPEEVDETSAEDEGISQR	0.658111221	5.0858
Q8K1S3	47.286	VYNpSSpTIGpSGSGIADGADIIGVIPPGpTpYPGDFSR	0.657289339	0.18689
Q9WUH1	140.93	TDpSPIPIEEASTPPGK	0.654278985	1.2356
Q8C8U0	157.91	DIGQpSNSDIDMPFAK	0.636537237	1.1044

Q8BTI8	105.46 SIpSYpSPVER	0.626802056	0.53083
E9QM77	107.57 TNpSPSIpSPSMISNAEHK	0.6114712	0.65252
Q3TWW8	133.89 pSMpSPPPK	0.610277066	0.79456
F7BGY9	156.87 SSSDSVEEETVDpSDTPPVIEK	0.597193192	0.69275
P14873	141.1 SDIpSPIpTPR	0.595663569	1.1207
Q7TPW1	189.87 EMIApSDDEEESpSPKIEK	0.587613116	1.0032
E9Q7E9	109.66 EPSGQPEDpSPEAETSTIDVFTEK	0.568472514	0.54167
Q8R1X6	71.08 KpSPEQESVSTAPQR	0.56679703	0.66786
Q6PHZ2	111.77 KPDGVKEpSTESSNpTTIEDEDVK	0.565706851	0.72569
P17095	129.29 KIEKEEEEGISQEpSpSEEEQ	0.563539025	1.0993
P15702	109.03 GEEEPIVGpSEDEAVEpTPTSDGPQAK	0.544040041	1.0226
Q80XU3	177.02 VVDYSQFQEpSDDADEDYGR	0.510594843	1.0693
Q99LJ0	85.457 DIpSPTIIDNSAAK	0.492077551	0.79678
Q5SW19	156.14 AVEDM(ox)GpSPQTAK	0.489188925	0.80587
P51859	77.776 AGDVIEDpSPKRPK	0.468999156	0.98894
E9Q6E5	63.624 RPTEAVpSPK	0.464187903	0.75161
P53986	172.6 AAQSPQQHSSGDPTEEEpSPV	0.462812977	0.99505
Q99KG3	205.78 GIVAAYSGEpSDpSEEEQER	0.449438202	1.3331
Q64012	105.22 IPAPQEDpTApSEAGpTPQGEVQTR	0.447027269	1.1427
Q68FF6	103.03 HGSGADSDYENpTQSGDPIIGIEGK	0.443262411	0.42931
P27546	200.98 AIETM(ox)AEQTTDVVHpSPSTDTTPGPDTEAAIAK	0.442948264	1.1003
P27546	70.555 AAEQMSTIPIDAPpSPIENIEQK	0.432675666	0.85165
Q8CH25	113.25 DVQDAIAQpSPEK	0.412609341	0.91613
Q8K019	102.52 EVQpSPEQVK	0.409584272	0.88292
Q8C1D8	100.67 ETTVApSDpSEEEAGKEESSVK	0.407896884	0.89946
P27546	138.05 DVAPPM(ox)EEEIVPGNDTpTSPKETETTIPIK	0.406685916	0.81678
Q9DAZ9	99.891 VTIQDYHIPDpSDEDEETAIQR	0.404220057	1.3974
Q9CZH7	111.34 VAEPEEpSEAEEPAAEGR	0.399808092	0.91715
P26645	131.43 EAAEAEPAEPpSSPAAEAEGASASSTSSPK	0.393313668	1.3318
Q62523	66.056 pSPGGPGPITIK	0.389833151	0.90923
Q569Z6	90.108 MDpSFDEDIAR	0.386817268	0.66547
Q60598	115.13 ASAGHAVpSITQDDGGADDWETDPDFVNDVSEK	0.376194417	1.0264
Q3UMU9	193.19 GGpSpSGEEIEDEEPVKK	0.361428365	0.60318

Q52KI8	143.39 KETEpSEAEDDNIDDIER	0.358602883	0.59538
E9QN88	156.17 VESTSVGSIpSPGGAK	0.356989861	0.68887
Q3UZ39	115.71 EIpSPVGEK	0.338879664	0.82605
P27546	185.95 DM(ox)pSPIPESEVTIGK	0.337906332	1.0598
P26645	145.55 VNGDApSPAAAEPGAK	0.33060037	0.94083
D3Z024	119.62 TASRPEDTPDpSPpSGPSSPK	0.323939099	0.96207
Q5NCR9	99.603 GAQEAEENPDADREFDDEpSpSEDGEKR	0.312656328	0.94814
Q99JT2	112.96 ESNPHPEWSFpTpTVR	0.308394498	0.10209
P14873	162.42 DVMSDETNNEETEpSPSQEFVNITK	0.287546367	0.94158
P27546	228.38 DMpSPSAETEAPIAK	0.287422396	0.92596
P07901	124.76 ESDDKPEIEDVGpSDEEEEEKK	0.262501641	0.76904
P14873	95.666 TPGDFNYAYQKPENAAGpSPDEEDYDYESQEK	0.250018751	0.80058
P62908	110.08 DEIIPTTPIpSEQK	0.229858637	1.2173
E9QA63	157.9 INSQEpSDEEPQISDVPHISK	0.212143069	0.74892
Q7TQH0	212.72 EVDGIITSDPMGpSPVSSK	0.198921844	0.92844
Q6PAM1	216.29 EQGVESPGAQPASpSPR	0.197009397	0.70159
Q91YE7	213.69 GIVAAYSGDpSDNEEEIVER	0.167706447	0.66063
Q8CI71	91.414 SAYQDYDpSDSDVPEEIKR	0.105987218	0.056415

*Before each phosphopeptide is listed the PTM score. Phosphorylated residues are indicated with a "p" and oxidated methiones with "(ox)".

Table S4 – D-domains in the *in vivo* substrates.

Substrate	Site	Sequence	Score	Percentile
Ahnak	V163	RRVTAYT**V**DVTGREG	0.5811	0.190 %
	V649	VKGEGLD**V**NVTLPEG	0.5631	0.135 %
	V1071	PKVSLPD**V**DLDLKGP	0.5712	0.158 %
	V1718	KLKSGVD**V**SLPKVEG	0.5532	0.120 %
	L2952	PKISMPD**L**NLNLKGP	0.5712	0.158 %
	I3026	PKISMPE**I**DLNLKGS	0.5404	0.094 %
	I4040	PKISMPE**I**DLNLKGP	0.5404	0.094 %
	V4376	PKISMPE**V**DLNLKGP	0.5404	0.094 %
	V4720	PKLGGGE**V**DLKGPKV	0.5451	0.103 %
Iws1	L535	KKPALKK**L**TLLPTVV	0.4671	0.021 %
Grp78*	I53	FKNGRVE**I**IANDQGN	0.6834	0.967 %
	V171	KKVTHAV**V**TVPAYFN	0.6670	0.748 %
	I221	KREGEKN**I**LVFDLGG	0.6816	0.943 %
	I360	KKSDIDE**I**VLVGGST	0.6247	0.381 %
Prdx6	L71	RNVKLIA**L**SIDSVED	0.5482	0.109 %
	I112	DKGRDLA**I**LLGMLDP	0.5224	0.070 %
	V189	KKGESVM**V**VPTLSEE	0.5078	0.052 %
Ranbp2	I719	RKTRDYL**I**RILDDSD	0.5811	0.190 %
	I1236	RREQVLK**I**CANHYIS	0.4596	0.017 %
	V1920	RREQVLK**V**CANHWIT	0.4596	0.017 %
	L2217	RRDQVLK**L**CANHRIT	0.4515	0.014 %

*D-domains for Grp78 meet the Medium Stringency criteria in Scansite. For all other substrates, the D-domains meet the High Stringency criteria.
